# Spike and burst coding in thalamocortical relay cells

**DOI:** 10.1371/journal.pcbi.1005960

**Published:** 2018-02-12

**Authors:** Fleur Zeldenrust, Pascal Chameau, Wytse J. Wadman

**Affiliations:** 1 Department of Neurophysiology, Donders Institute for Brain, Cognition and Behaviour, Radboud University, Nijmegen, the Netherlands; 2 Cellular and Systems Neurobiology, Swammerdam Institute for Life Sciences, University of Amsterdam, Amsterdam, the Netherlands; University College London, UNITED KINGDOM

## Abstract

Mammalian thalamocortical relay (TCR) neurons switch their firing activity between a tonic spiking and a bursting regime. In a combined experimental and computational study, we investigated the features in the input signal that single spikes and bursts in the output spike train represent and how this code is influenced by the membrane voltage state of the neuron. Identical frozen Gaussian noise current traces were injected into TCR neurons in rat brain slices as well as in a validated three-compartment TCR model cell. The resulting membrane voltage traces and spike trains were analyzed by calculating the coherence and impedance. Reverse correlation techniques gave the Event-Triggered Average (ETA) and the Event-Triggered Covariance (ETC). This demonstrated that the feature selectivity started relatively long before the events (up to 300 ms) and showed a clear distinction between spikes (selective for fluctuations) and bursts (selective for integration). The model cell was fine-tuned to mimic the frozen noise initiated spike and burst responses to within experimental accuracy, especially for the mixed mode regimes. The information content carried by the various types of events in the signal as well as by the whole signal was calculated. Bursts phase-lock to and transfer information at lower frequencies than single spikes. On depolarization the neuron transits smoothly from the predominantly bursting regime to a spiking regime, in which it is more sensitive to high-frequency fluctuations. The model was then used to elucidate properties that could not be assessed experimentally, in particular the role of two important subthreshold voltage-dependent currents: the low threshold activated calcium current (*I*_*T*_) and the cyclic nucleotide modulated h current (*I*_*h*_). The ETAs of those currents and their underlying activation/inactivation states not only explained the state dependence of the firing regime but also the long-lasting concerted dynamic action of the two currents. Finally, the model was used to investigate the more realistic “high-conductance state”, where fluctuations are caused by (synaptic) conductance changes instead of current injection. Under “standard” conditions bursts are difficult to initiate, given the high degree of inactivation of the T-type calcium current. Strong and/or precisely timed inhibitory currents were able to remove this inactivation.

## Author summary

Neurons in the brain respond to (sensory) stimuli by generating electrical pulses called ‘spikes’ or ‘action potentials’. Spikes are organized in different temporal patterns, such as ‘bursts’ in which they occur at a high frequency followed by a period of silence. Bursts are ubiquitous in the nervous system: they occur in different parts of the brain and in different species. Different mechanisms that generate them have been pointed out. Why the nervous system uses bursts in its communication, or what type of information is represented by bursts, remains largely unknown. Here, we looked at bursting in thalamocortical relay (TCR) cells, neurons that form a bridge between early sensory processing and higher-order structures (cortex). These cells fire bursts as a result of the activation of two distinct subthreshold ionic currents: the T-type calcium current and the h-type current. We investigated experimentally and computationally what features in the input makes TCR cells respond with bursts, and what features with single spikes. Bursts are a response to low-frequency slowly increasing input; single spikes are a response to faster fluctuations. Moreover, bursts are rare and highly informative, in line with an earlier hypothesis that bursts could play a ‘wake-up call’ role in the nervous system.

## Introduction

Neurons in the nervous system respond to (sensory) stimuli by firing action potentials in a more or less regular way. Some neurons can also fire groups of related action potentials with a relatively high rate, followed by a period of silence. This firing mode is called bursting. Burst-firing has been described in many different species and systems, from the CA3 region of the rodent hippocampus [1] to the electrosensory system of the weakly electric fish [2].

Mammalian thalamocortical relay (TCR) cells can switch between tonic and burst firing [3],[4]. Bursts in TCR cells are generated by an after-hyperpolarization rebound, due to the removal of inactivation of the low-threshold (T-type) calcium current [5, 6], sometimes called a low-threshold spike, and often in concert with the hyperpolarization activated h-type current. The functional role of these bursts and what they code for in the input signal remains, however, unknown.

The burst mode of TCR cells has traditionally been taken as an indicator of slow-wave sleep and pathological conditions, because it prevents correct execution of the relay function of the thalamus [7]. Later it was found in awake animals that TCR cells can, however, combine burst firing with effective relay properties([8], for a review see [9]). Theories about the function of bursts in this system range from containing the same information as single spikes [8], to a “wake-up call” function [9], suggesting that bursts are often more than just an artefact of cellular properties. Bursts do also have a larger impact on their target cells than single spikes [10, 11] and are a more reliable way to propagate a message.

The key question in this paper is: what information in the input signal is represented by single spikes and what information is represented by bursts in the TCR output spike train and can we pinpoint the cellular mechanisms that are responsible for their respective generation? The neuron is considered as an (active) ‘filtering device’, that responds to salient features of the input, and we determine these salient features. Our study combines an experimental with a modeling approach: experimentally, Gaussian frozen noise was injected into the somata of rat TCR cells kept under current-clamp conditions *in vitro* to determine what features in the input the cells responded to. We investigated how this neural code is influenced by the overall background (‘membrane state’) and by the regime (bursting/spiking) the neuron is in. The results were used to adjust, validate and corroborate a TCR computational model cell [12]. At least two hypotheses that could not be addressed experimentally, were then investigated in the model: 1) Are T-type calcium current and h-type current contributing in a specific way to the initiation of single spikes and bursts? and 2) Do our conclusions also hold in the high-conductance state, which closer resembles the *in vivo* condition?

## Results

### Spike trains

**Fig 1:**
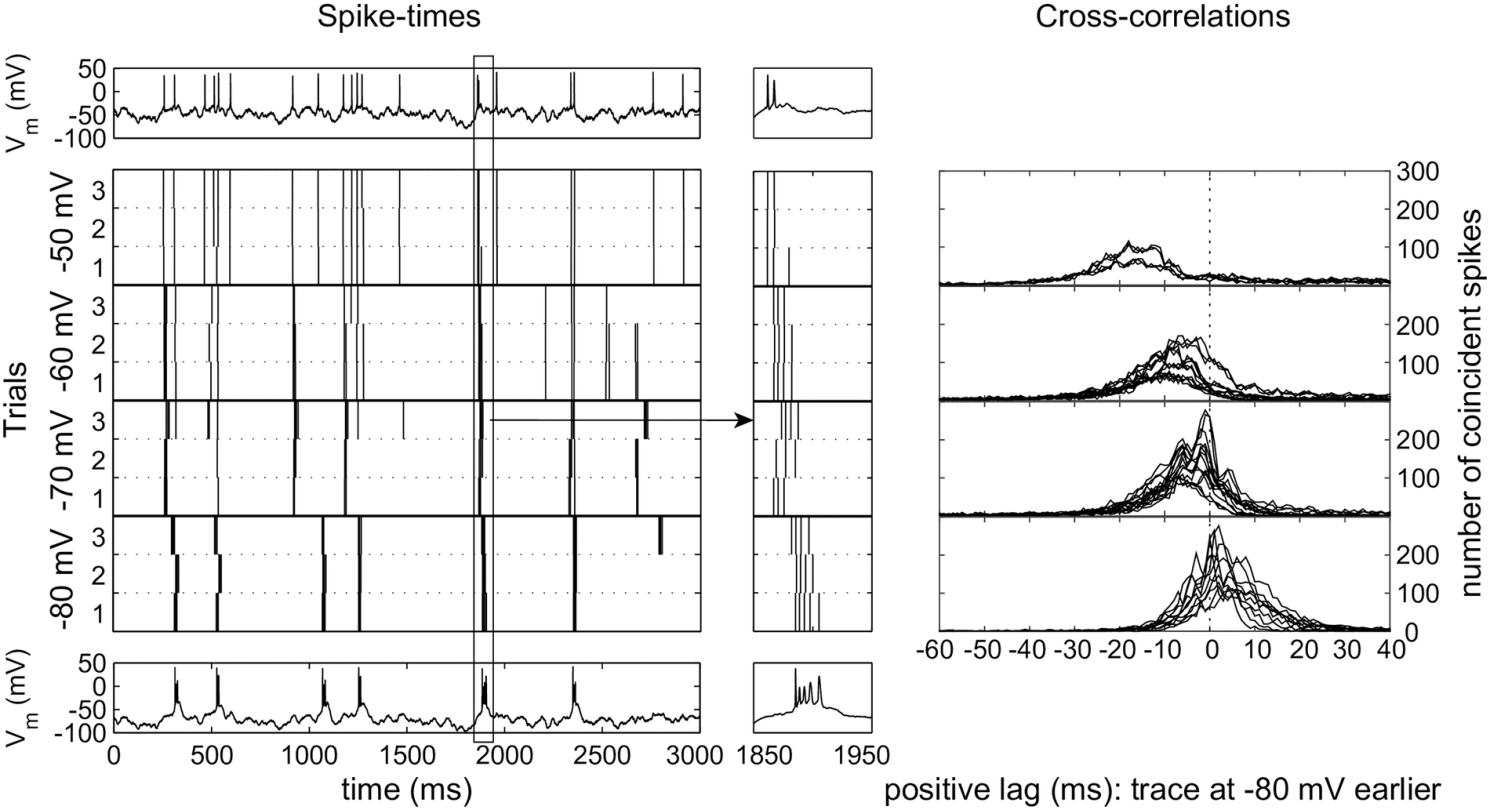
*In vitro* TCR neuron response to injected frozen noise. Left panel: Spike trains induced in a TCR cell by frozen noise with identical amplitude and various DC offsets; trace is a 3 s extract from a standard 300 s recording. Top: example trace at −50 mV. Bottom: example trace at −80 mV. Vertical bars in the panels in between mark the detected spikes in the 3 recorded replicates at 4 levels of depolarization, from top to bottom: −50, −60, −70 and −80 mV. The neuron responds earlier at more depolarized membrane potentials. Middle panel: 100 ms zoom of a single event starting at 1850 ms (zoom indicated in the left panel). Top and bottom show the event at −50 mV and −80 mV. Right panel: Cross-correllograms between a reference spike train at −80 mV and the spike trains obtained in the same cell at the different membrane states. Recordings from 5 cells (=15 traces), resulting in 10 comparisons at −80 mV, 15 comparisons at −70 and at −60 mV; only 2 cells gave sufficient results at −50 mV (6 comparisons). On depolarization the peaks shift to the left, indicating that the neurons fire earlier.

TCR neurons respond in a characteristic, strikingly reproducible way to the injection of frozen noise (Fig 1, left panels), suggesting that they respond to specific features in the input and (in the slice) are subjected to little intrinsic noise. The response is voltage dependent as it changes when the neuron is depolarized to different voltage levels, which we will refer to in the rest of the paper as membrane states. Upon depolarization, neurons shift from a bursting to a spiking regime, and they respond earlier (Fig 1, middle panels). To compare the different spike trains, we took one of the spike trains at a membrane state of −80 mV as a reference and calculated the cross-correlogram with all other spike trains recorded in that neuron. Indeed, the neuron spikes up to 20 ms earlier in time when the membrane potential is around −50 mV than when it is around −80 mV (Fig 1, right panels). This result is quite robust, as it held for all cells (*n* = 5) and all repetitions (three per cell).

### Burstiness

**Fig 2:**
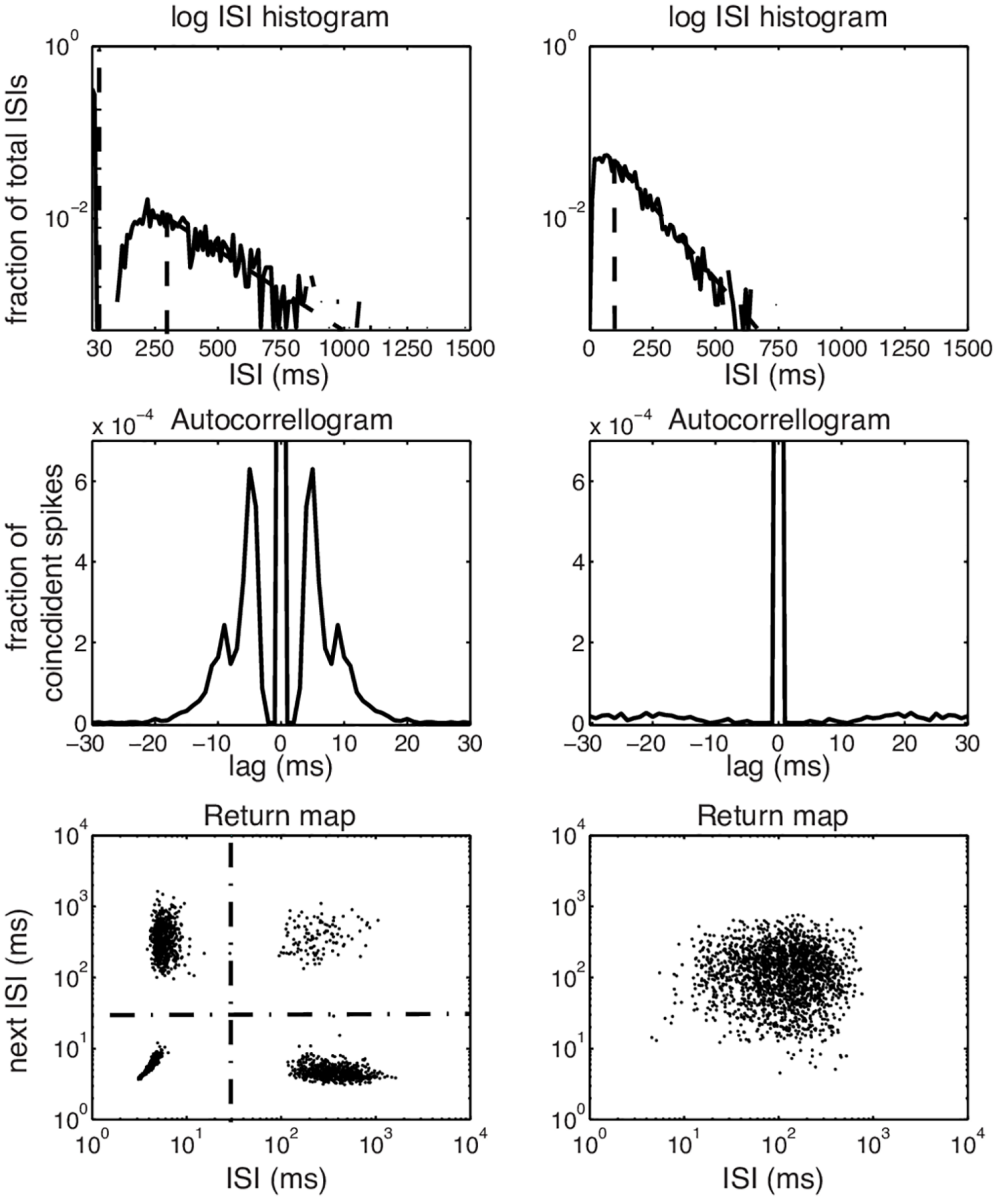
Quantification of burstiness in a TCR cell spike train. Left panels: Spike train recorded at a membrane state of −80 mV. The ISI histogram (top row) shows a first peak around 5 ms and a second one around 250 ms. The tail decays exponential (dashed line) above 300 ms; so events were considered independent if further apart than 300 ms. The Autocorrelogram (middle row) shows peaks at 5 and 10 ms. In the return map (bottom row) ISI n is plotted against ISI n+1; k-means clustering of this data provides 4 clusters and a silhouette value of 0.96. Based on the ISI histogram and the return map we used an interval of 30 ms to separate single spikes from bursts. Right: Spike train recorded at a membrane state of −50 mV. The ISI histogram (top row) indicates a single Poisson process with events independent if they were further apart than 100 ms. The autocorrelogram (middle) shows only one central peak around zero and the return map (bottom) shows an unimodal structure confirmed by a silhouette value of 0.48.

A neuron that only responds to the occurence of a certain feature in the input and that is not, for instance, an intrinsic oscillator, responds to Gaussian input with a spike train that, like a spike train generated by a Poisson process, has an exponential ISI distribution. When on the other hand small intervals are over-represented, it is likely that at least a fraction of the events consists of bursts of action potentials: short periods of time with a substantially higher firing rate (up to 100 – 200 Hz). There are various techniques to identify the presence of bursts in the spike train: 1) the interspike interval (ISI) distribution shows a distinct peak at a small interval time (Fig 2, top row) [2]), 2) the autocorrelogram of the spike train shows a distinct non-zero peak (Fig 2, middle row) [13]) and 3) the return map, that plots successive spike intervals against each other (Fig 2, bottom row) [8]), should demonstrate clear clustering. We combined the three methods and came to an optimal separation between spikes and bursts in TCR cells at an interval time of 30 ms. Events with an exponentially decaying ISI distribution can be assumed independent [14]. We determined the minimal ISI for which events can be assumed to be independent, by fitting an exponential curve (Fig 2) to the tail of the distribution, and determining by eye where the distribution started to deviate from the exponential distribution. This restriction is stronger for bursting regimes (about 300 ms) than for spiking regimes (about 100 ms) (Fig 2, top row: dashed lines). At a membrane state of −80 mV, the neuron was in a bursting regime: the majority of events were classified as bursts, whereas at a membrane state of −50 mV the neuron is in a spiking regime: most events consist of isolated single spikes. For membrane potentials between these two values the neurons were in a mixed regime, in which they responded with both spikes and bursts. Bursts typically ride on a short low-threshold spike (LTS) that is about 10 mV above mean background (Fig 1, middle column, bottom row for a typical example).

### Transfer function

**Fig 3:**
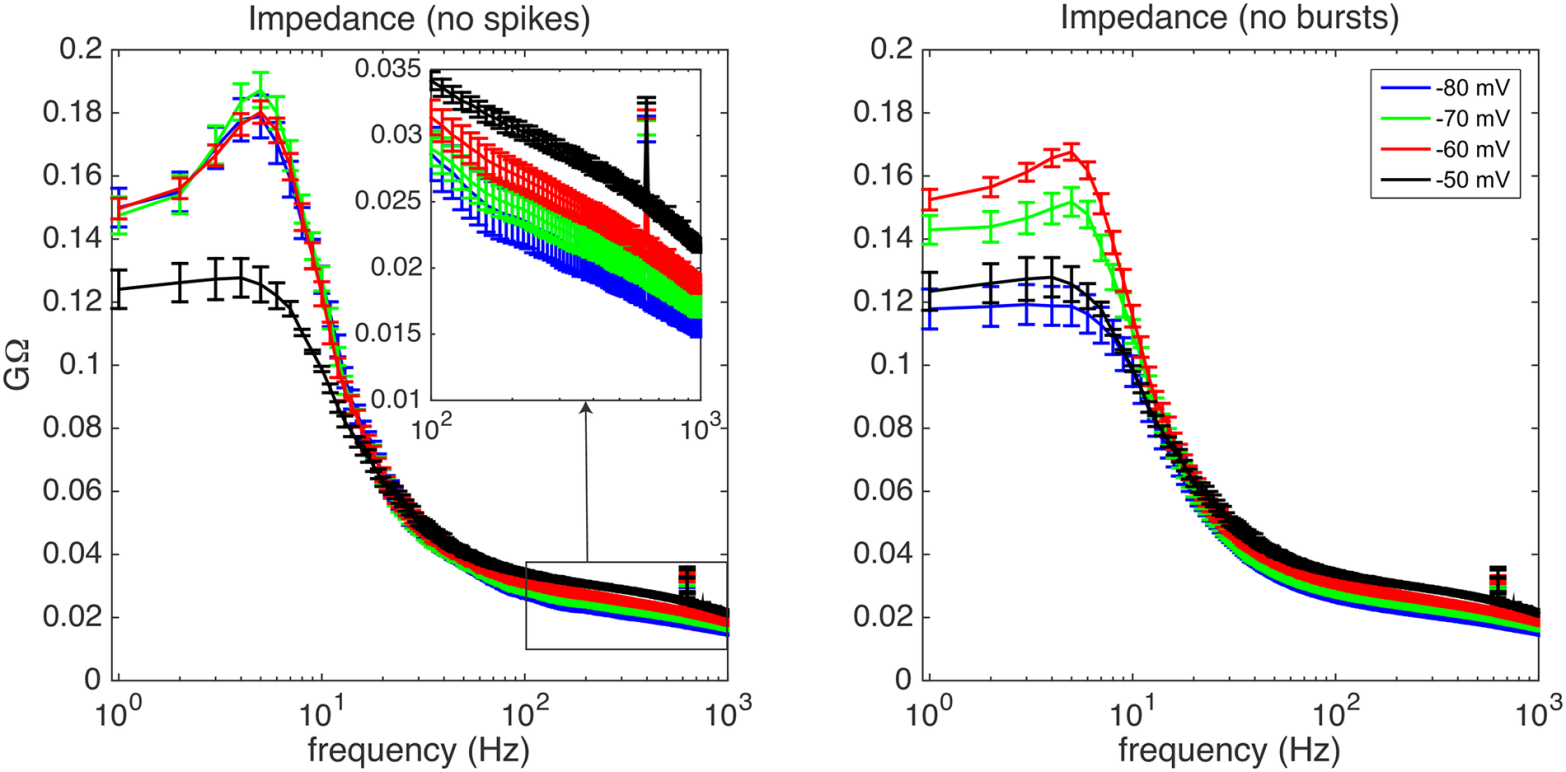
Transfer function of a TCR neuron *in vitro*. Impedance of the TCR neuron calculated as the amplitude of the transfer function between current input and voltage output for the four defined membrane states (blue: −80 mV, green: −70 mV, red: −60 mV, black: −50 mV). Left: A small time window was cut out and interpolated around each single spike to avoid contamination of the spectra by the fast spikes. Inset: zoom with one in ten data points on the frequency range between 100 and 1000 Hz. Right: same data, but now with also a larger cut-out window around each burst including the LTS. Note the resonance peak around 5 Hz that is related to bursting: absent in the −50 mV trace and considerably reduced if we cut away the LTSs in the left panel. Error bars reflect standard error of the mean.

The (subthreshold) membrane of TCR cells acts as a high order low-pass filter with resonance. We characterized its transfer function (Fig 3): i.e. the amplitude of the ratio of the membrane potential trace (output) and the injected current (input) determined in the fourier domain [15]. The transfer function shows resonance for frequencies around 5 Hz, which is at least partialy caused by slow LTSs that accompany each burst: the resonance peak reduces substantially once we cut out a window around each burst (Fig 3, compare right and left panel). It is well known that in TCR cells at least two membrane currents underly this resonance: the low threshold calcium current *I*_*T*_ and the hyperpolarization-activated cyclic nucleotide—gated current *I*_*h*_ [16–19].

On depolarization to −50 mV, where bursts and resonance disappear, the filtering properties of the neuron become more broadband. The neuron’s time constant decreases, which implies that the (subthreshold) membrane potential can better follow the input current at higher frequencies. In additional experiments (Figure not shown), where the noise input was filtered with *τ* = 1 ms instead of *τ* = 10 ms which also results in a more flat spectral density, we observed the same phenomenon. For the cell population the precise value of the resonance frequency and the point of the steepest decrease in impedance varied slightly, because different neurons have slightly different LTS duration and may vary in membrane time constant.

### Frequency content, coherence and information transfer

**Fig 4:**
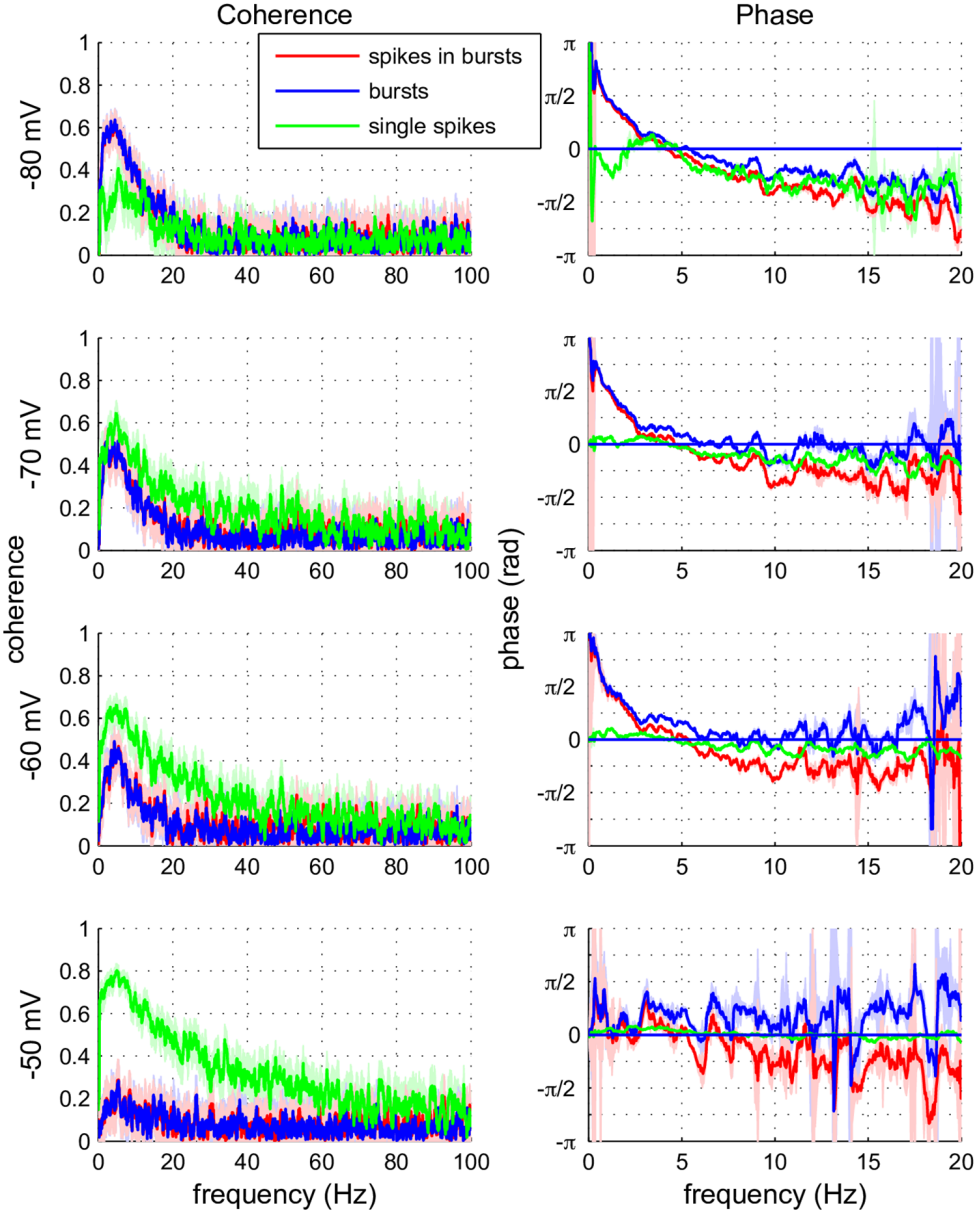
Coherence and phase between input current and spikes or bursts. Coherence (left column) and phase (right column) between the input current and the timing of three different activity components in the TCR cells: bursts, spikes and ‘follower’ spikes in bursts. Calculations were performed over the first 500 s of each trial and for the four different membrane states. Coherence decreases for higher frequencies. Bursts (blue traces) phase-lock mainly to low frequencies, with positive phase (upstroke). Isolated spikes (green traces) show more broadband phase locking. Spikes in a burst (red traces) seem to follow the properties of the burst to which they belong. The colour shade around a trace indicates the confidence interval (95%) based on a jackknife method.

To further elucidate the difference between spikes and burst in TCR neurons, we investigated to what frequencies in the input the neurons respond, both sub- and suprathreshold. The coherence (calculated with the help of the Chronux toolbox (http://chronux.org/, [20]) between the different types of events and the input shows a complementary picture to the transfer function: bursts phase-lock with positive phase (upstroke) to low frequencies, while phase decreases at higher frequencies (Fig 4). A similar pattern arises for more depolarized potentials. At a membrane state of −50 mV the phase of the first and following spikes in a burst does not sufficiently phase-lock to extract a consistent phase value: there are simply not enough bursts. However, spikes show more broadband behaviour and phase-lock to the peak of the oscillation (phase = 0 rad), except at a membrane state of −80 mV, where bursts dominate. In conclusion, at a hyperpolarized membrane state the neuron is basically a low-pass filter with resonance around 5 Hz. On depolarization the resonance disappears and the filtering properties of the neuron become more broadband.

**Fig 5:**
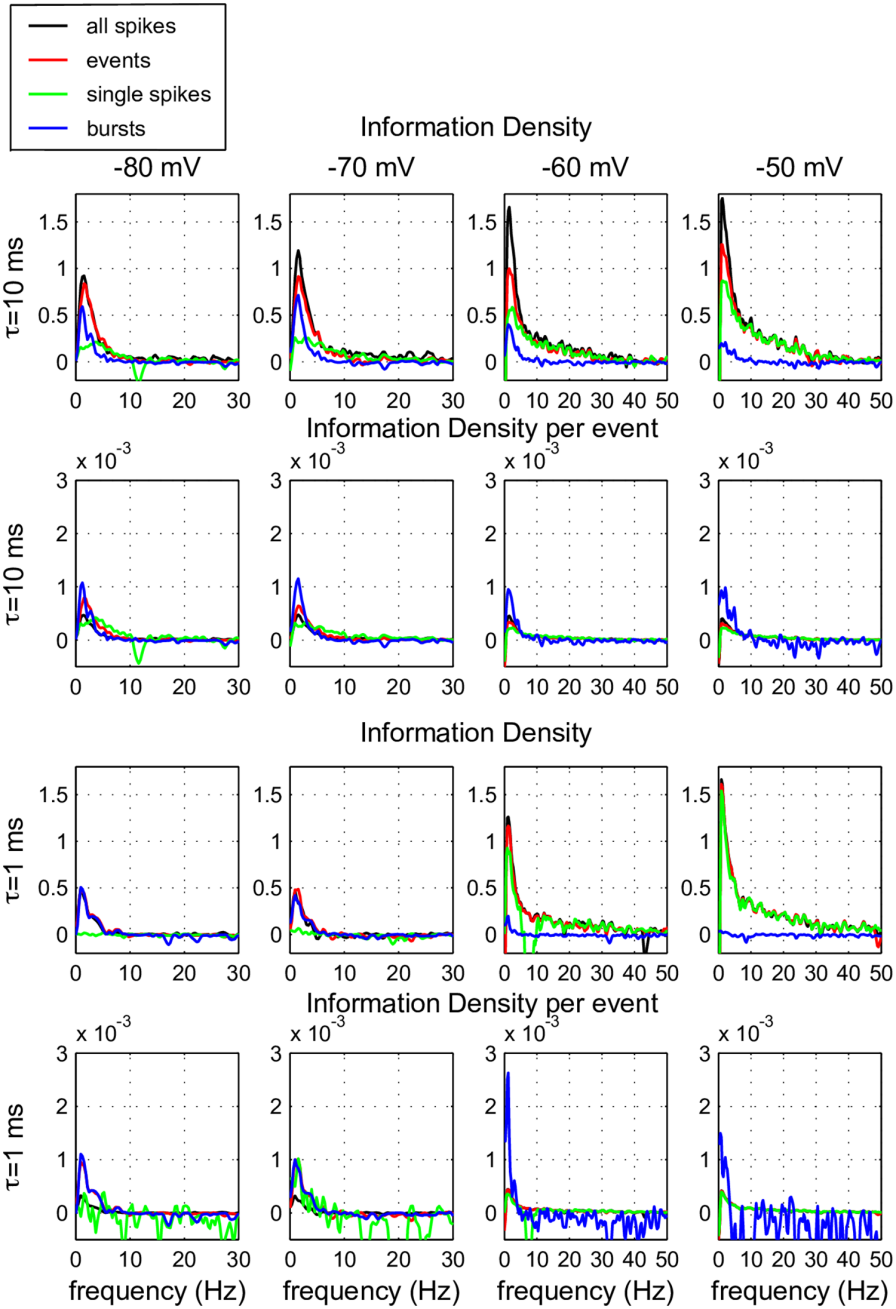
Information density carried in the TCR output signal. Typical example of the information density as a function of frequency calculated for several relevant components in the output signal of a TCR cell at the four different membrane states (left to right as defined in Fig 3). Two different input signals were used, both contained the same frozen Gaussian noise, filtered with an exponential filter with a time constant of either *τ* = 10 ms (top two rows) or *τ* = 1 ms (bottom two rows). We calculated the information density for each isolated spike (green), each burst (blue), each event (red = either isolated spike or burst) and all spikes (black, isolated or contained in a burst). The information density was calculated either per type of activity (row 2 and 4) or over the full trace (row 1 and 3).

The information transfer to the output spike train of the TCR cell at the four different membrane states was calculated following [8, 21, 22] for all activity components: bursts (blue traces), isolated spikes (green traces), events (either a burst or an isolated spike, red traces) and all spikes (whether isolated or in a burst, black traces). The information transfer was calculated for the overall output spike train as well as for each component class (Fig 5). To assess a possible bias, each calculation was repeated 50 times for Poisson spike trains with the same number as events (S4 Fig). Most information was transferred in the frequency band up to 10 Hz, making the TCR neuron effectively a low-pass filter. The input current was low-pass filtered with a *τ* = 10 ms exponential filter, because TCR neurons respond stronger to low-frequency components of the input. Therefore, with a low-pass filtered input, the amplitude of the stimulus can be kept low, which increases the cell-life in an *in-vitro* setup. However, to assess the possible high-frequency components in the information transfer of these cells, we have repeated the experiments with input filtered with an *τ* = 1 ms exponential filter, so the input contained less power in the low frequency band and more power in the high frequency band. Indeed, in Fig 5 (compare first and third row) it is shown that the neuron mostly responds to low frequencies in the input. For the *τ* = 1 ms input, the neuron responds with a lower frequency, and therefore transfers less information, especially at the hyperpolarized membrane state. The amount of information per event does not depend on the used time constant (Fig 5, compare second and fourth row), suggesting that in the *τ* = 10 ms case the neuron transfers more information by simply responding with more events.

Comparing the traces for all spikes (black) with the traces for events (red), so without the “follower-spikes” in bursts, reveals that there is some, but not much, information carried by the follower “spikes in bursts”. For *τ* = 1 ms the traces practically overlap (third row), and the information per event decreases if the “spikes in bursts” are taken into account (fourth row), suggesting that they contained little information. For *τ* = 10 ms there is some low frequency information in the “spikes in bursts” (top row), but the information per event decreases when “spikes in bursts” are taken into account, confirming our conclusion that “spikes in bursts” carry little information. Observations on all other cells led to the same conclusions.

The traces for isolated spikes (green) and bursts (blue), illustrate that bursts are never capable of transferring information in frequency bands higher than about 6 Hz (but see Discussion). Single spikes on the other hand, can transfer information up to 50 Hz. At hyperpolarized holding potentials bursts carry most of the information in the spike train. Bursts also carry more information per event (second and fourth row left). Only if there is enough power in the frequency band of about 6 – 10 Hz, information is carried by single spikes (first and second row left). Depolarizing the cell gives the advantage to single spikes (first and third row), even in low frequency bands, since they increase their number, but the information per spike does not increase (second and fourth row). At depolarized potentials bursts are less abundant, but they stay highly informative (second and fourth row). So in mixed regimes bursts may be rare and report only about the low input frequencies, but they are highly informative. Single spikes are less informative per event, but they carry most information in the spike train and they carry all high-frequency information.

### Reverse correlation

Reverse correlation can be used to determine the features in the input signal to which neurons respond [23–26]. However, care has to be taken to prevent interactions between successive events from interfering with the analysis [14, 27]. Therefore we restricted our analysis to independent events and started by excluding spikes inside bursts. In addition the more ‘bursty’ a neuron is, the longer the events influence each other (see above). As we observed that burstiness is state dependent, it is likely that the inter-spike interval at which events can be considered independent also changes with the membrane regime: for the membrane state at −80, −70, −60 and −50 mV, we used minimum inter-spike intervals of respectively 300, 250, 200 and 100 ms. In experiments where the exponential filter with *τ* = 1 ms was used, we increased these values to compensate for the longer observed integration time of the neuron under that condition (figures comparable to Fig 2 not shown for this condition). We show here the results from a single example cell, since the main results did not differ much between cells. To assess the variability between cells, we show the reliability of spike timing within cells, between cells, and between recordings in S1 Fig. In S2 Fig we compare the ETAs obtained from different cells. Even for low reliability, the ETAs are still relatively similar.

#### Event-Triggered Average (ETA)

**Fig 6:**
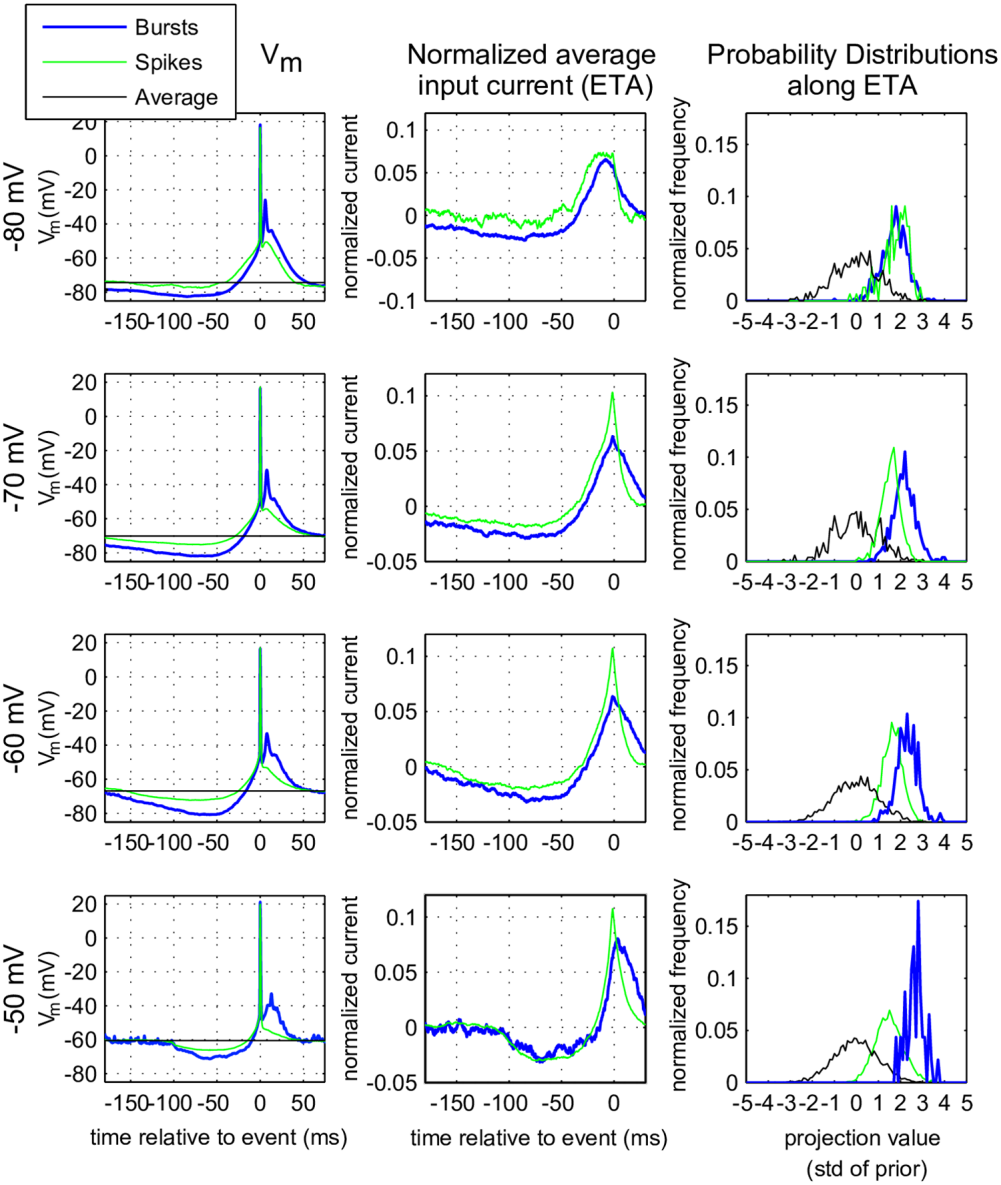
Event Triggering Average (ETA) of membrane potential and injected current. Left column: ETA of the membrane potential 200 ms preceeding single spikes (green),aligned at t = 0, or bursts (blue). The peak of the spike (or first spike in the burst) was used as trigger; this implies perfect coincidence of isolated spike and first spike in the burst; later spikes in the burst tend to average out. The horizontal black lines indicate the mean value of the subthreshold membrane potential calculated from the entire trial. Middle column: ETA of the input current (mean value substracted and normalized with the L2 norm) using the triggerpoints from the voltage (peak of the spikes, left column). Right column: probability distributions of a convolution of the normalized ETA with event-triggered (blue/green lines) or with a random input (black line, also called the prior). Rows indicate membrane states.

In Fig 6 the Event-Triggered Averages over both the membrane potential (left column) and the input current (middle column) are shown for isolated spikes (green) and the first spike in each burst (blue) at the four different membrane states. Bursts need a slow hyperpolarizing input about 200 to 30 ms before the event, followed by a strong depolarizing input that starts about 30 ms before the spike, and continues until about 30 ms after the spike. With increasing depolarization, the event shifts from right after the peak of this positive wave to the top and even to before the top. This implies that spikes in a burst ride on a distinct depolarizing wave and can often be triggered before the wave reaches its maximum. In the spiking regime, at a membrane state of −50 mV, a strong depolarization that continues after the first spike of a burst is needed in order to initialize a burst.

**Fig 7:**
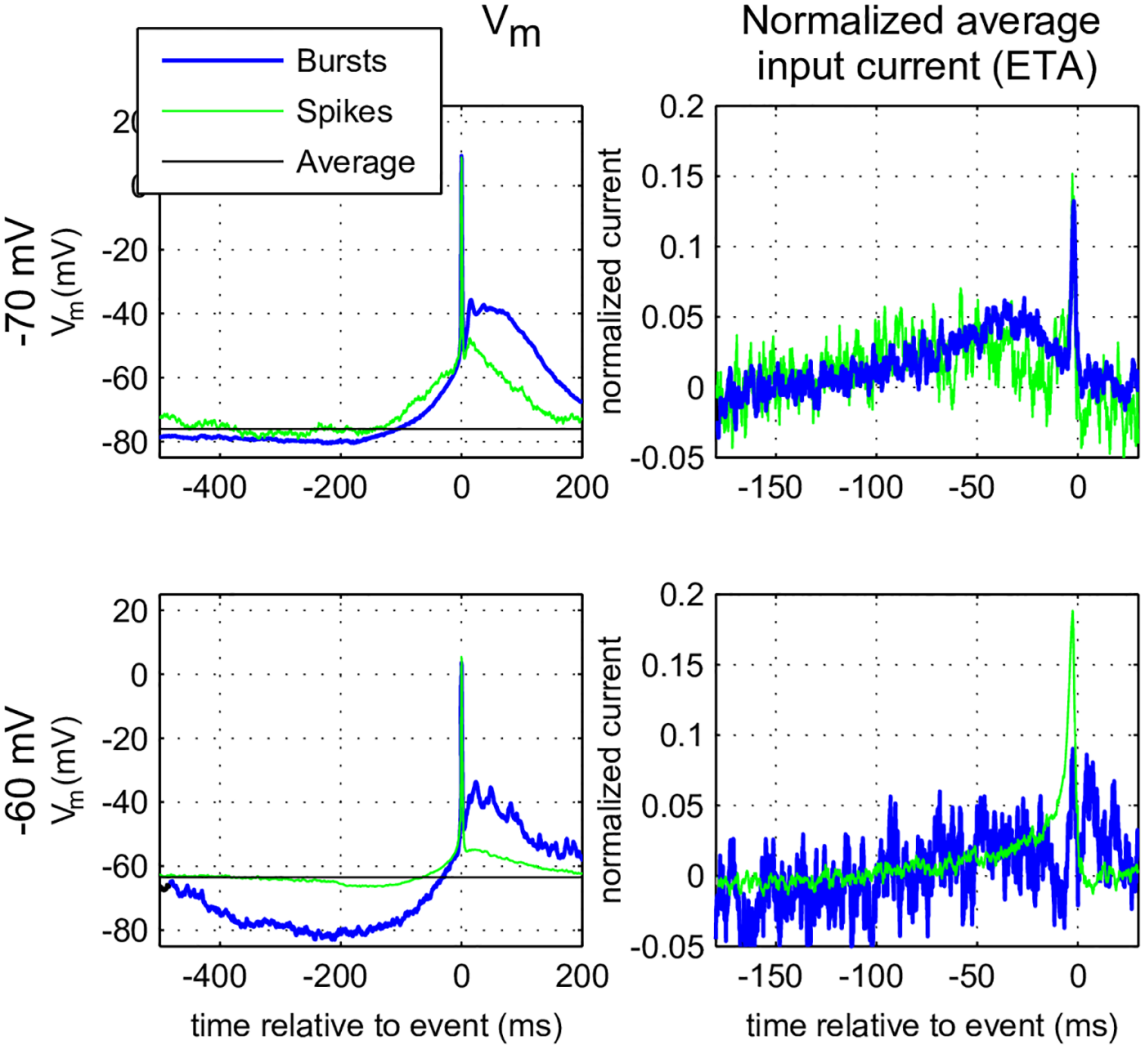
Event Triggering Average (ETA), using a fast-fluctuating input current. Repeat of the experiment in Fig 6 but now with the input filter (*τ* = 1 ms) and only for mixed membrane states at −70 mV (top row) and −60 mV (bottom row) as only they produced sufficient spikes *and* bursts for the analysis. Left column: ETA of the membrane potential 500 ms preceeding single spikes (green) or bursts (blue). Right column: ETA over the input current (mean value substracted and normalized with the L2 norm). The horizontal black line indicates the mean value of the subthreshold membrane potential calculated from the entire trial. Other details as in Fig 6.

Single spikes need less of the slow hyperpolarization before the event, and less depolarization during/after the event than bursts. The distributions (Fig 6, right panels) show that in all but the −80 mV regime, bursts (blue) have a higher threshold (distribution is shifted to the right) than single spikes (green), suggesting that bursts are more selective events, they are “harder to initiate”. The hyperpolarization needed to initiate an event at depolarized membrane potentials is shorter than the one needed in the more hyperpolarized states. At the same membrane state the peak in the ETA for single spikes is sharper (Fig 6, bottom middle panel, green trace), suggesting that the integration time of the neuron is smaller. This was further investigated by determining the ETA after injecting noise exponentially filtered with *τ* = 1 ms (Fig 7). This broadband input triggers fewer bursts because there is less power in the low frequency band that is most efficient for burst generation (see previous section). The longer integration time for the stimulus with *τ* = 1 ms discussed above can also be observed in Fig 7. The reduced number of events make ETAs harder to construct and much noisier for this condition. Nevertheless Fig 7 suggests that the neuron needs processes at two different time scales in order to respond: 1) a fast depolarization, in the order of a few milliseconds, seen as an upswing of both the ETA and the average membrane potential right before the event and 2) a longer lasting hyperpolarization in the order of hundreds of milliseconds. When the input current is filtered with *τ* = 10 ms this distinction is less pronounced. At depolarized membrane potential, less long-lasting hyperpolarization is needed to trigger a spike, which explains how the neuron becomes capable of transmitting more information at higher frequencies.

In conclusion: in mixed regimes bursts are a response to slow long-lasting (hundreds of milliseconds) hyperpolarizations followed by a strong depolarization, which is boosted by the neuron itself (depolarizing after potentials or DAP), whereas single spikes are a response to much faster features with a lower threshold. On depolarization the integration time of the TCR neuron decreases. In very hyperpolarized states even single spikes need slow long-lasting hyperpolarizations for their initiation.

#### Event-Triggered Covariance

**Fig 8:**
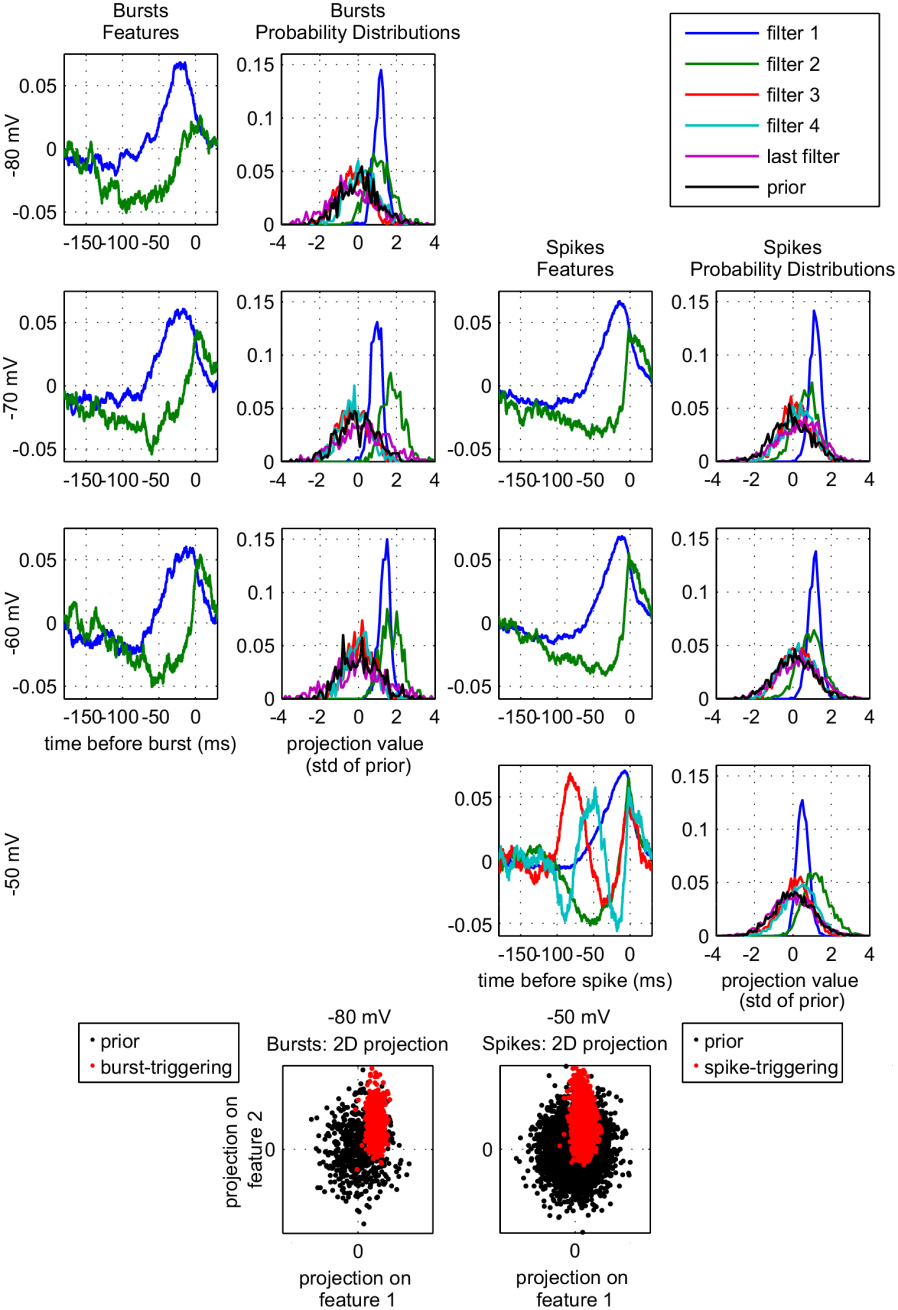
Event Triggered Covariance (ETC) of membrane potential and injected current. ETC was calculated for bursts (left two columns) and isolated spikes (right two columns) for four different membrane states (top four rows). ETC needs far more realizations than ETA, so at −80 mV, where burst dominate, we can only give results for bursts. While at −50 mV, where bursts are rare and spikes dominate, we can only give results for spikes. Only filters for which the probability distribution was substantially different from the prior, are shown. In S3 Fig we show the corresponding eigenvalues. Bottom row: projections along the first two features for bursts at a membrane state of −80 mV (left) and for spikes at a membrane state of −50 mV (right).

To investigate the origine of spikes and bursts in more detail, we performed an Event-Triggered Covariance (ETC) analysis [23, 25, 26] on the input current. In particular, with the ETC analysis, a multi-dimensional event-triggering feature space can be determined, instead of a single ‘optimal’ filter. ETC analysis needs a large number of events (>1000) which implies long recordings, because the events also need to be independent in time. The longest traces that we could stably record for the lowest membrane state lasted 500 s. In the most depolarized state there were not enough bursts for an appropriate ETC analysis for bursts and in the most hyperpolarized state the lack of sufficient isolated spikes prevented the analysis for spikes (Fig 8). The relatively low number of events also explains why the probability distributions are quite noisy. Finally, decision functions (Materials and methods: Eq 9) should be calculated by dividing the event-triggering distribution by the prior distribution; in our case, the noise levels made these graphs not insightful). The left column of Fig 8 shows the filters resulting from the ETC analysis for bursts. The second column shows the distribution of the burst-triggering input projected onto those filters ($P({\vec{v}}_{i}|event)$) with the four smaller eigenvalues, with the largest eigenvalue (‘last filter’) and with the prior distribution ($P({\vec{v}}_{i})$). In the hyperpolarized membrane states(Fig 8, top left) bursts are selective for two features represented by the first two filters: a slow integrating one (blue trace) and a slow hyperpolarizing one (green trace). On depolarization (row 2 and 3), the bursts become more selective for the second feature (probability distribution shifts to higher projection values), and the feature itself changes to include a stronger and sharper positive part around t = 0, whereas the negative part becomes less prominent at earlier times. The projection values for the second filter appear to be further right for membrane potentials around −70 mV than for membrane potentials around −60 mV. However, in the current analysis it is difficult to compare the projection values between voltages, as the exact shape of the filters is also not consistent between voltages (albeit similar). So this can be a result of the change in shape of the second filter, of the filtering characteristics of the neuron, or to other parts of the analysis, but it does not affect our conclusions: on depolarization bursts become more selective for fluctuations, instead of only for hyperpolarization. The third and fourth column repeat this analysis for the spikes in the train. At hyperpolarized membrane states (Fig 8, middle rows) single spikes are selective for two features that look similar, be it somewhat sharper, to the features that trigger bursts. On depolarization this filter shows a higher peak around zero and the neuron becomes more selective for the fluctuating filter (probability distribution shifts to higher projection values), whereas the neuron becomes less selective for the integrating filter (blue trace). This reflects that single spikes are ‘easier’ to initiate (the single spike frequency becomes higher) and more and more a response to fast fluctuations. The additional features represented in the membrane state at −50 mV (Fig 8, row 4) show strong oscillations that do not attenuate, indicating that they code for silence [14]. The bottom panels of Fig 8 illustrate the two-dimensional projection of the event-triggering stimuli onto the first two filters for the conditions with the highest number of bursts (left) and single spikes (right). For these conditions, these two features do not seem to depend on each other (bottom left panel): the burst-triggered values (red dots) suggest a two-dimensional Gaussian distribution. For single spikes (bottom right panel) the first two filters show a little interdependence, in that there are fewer projections that are both negative, and the very strong projection onto the second filter is associated with a weaker projection onto the first filter. However, there were not enough events in all conditions to analyze this systematically.

In conclusion: the first two features are relatively stable across voltages and event type. This means that the changes observed in the ETA (Fig 6), are mostly due to a different linear combination of these two features, i.e. the cell samples a different region of the same subspace. On depolarization, the strongest changes are in the feature selectivity seen in projections. Spikes are more selective for fast fluctuations (second feature) than bursts, and they are less selective for integration (first feature). This distinction becomes more prominent with depolarization and explains the observed changes in the optimal filters in the previous section.

### Model

Although it is relatively well known which ionic currents in TCR cells contribute to firing and bursting behaviour [5, 6, 17, 18], it is very difficult to experimentally investigate their individual dynamic role without heavily interfering with the firing pattern. A better approach is to use a computational model that replicates the firing patterns and then computationally investigate the underlying contribution of various currents activated in the different regimes. We started with a well validated, published TCR cell model and tuned it in such a way that injecting our standard frozen noise signal resulted in spike and burst trains that resembled the experimentally obtained trains of action potentials and bursts at a millisecond time range: i.e. the reliability between the model spike train and the experimental ones did not differ more than the variation in the experimental spike trains. This puts a strong requirement on the model neuron. In addition we are highly interested in the ‘high-conductance state’ [28], which better approximates the *in vivo* situation, but is also very difficult to mimic in acute slice experiments.

#### Model description and tuning

In this study we used the well validated three-compartment model of Destexhe et al. [12] implemented in the NEURON simulation environment [29]. The morphology used in this model is a reduction to three compartments (soma, proximal and distal dendrites) of a reconstructed multi-compartment model of a rat dissociated TCR cell. We chose a three-compartment model, since it has been shown in several other neuron models [30], that the interactions between soma and dendrite play an important role in the generation of bursts. The spike train output of the model covers the basic properties of the TCR cell, such as burst generation caused by the low-threshold T-type calcium current (maximal conductance ${\bar{g}}_{T}$), a shift from a bursting to a spiking regime and earlier spiking with depolarization, but it had to be tuned with respect to a number of parameters and was slightly expanded. Firstly, the cells in our experiments had a smaller integration time than the original model, which can be seen from comparing the traces and from the ETAs. Secondly, the bursts produced by the cells in our experiments contained less spikes than observed in the original model, most likely because the ionic mechanisms that normally slow down burst-firing were not completely implemented. We added a high-threshold calcium current (with maximal permeability ${\bar{p}}_{L}$) and included a calcium-activated potassium current (maximal conductance ${\bar{g}}_{KC}$) [31] to the soma, which reduced the maximum firing rate of the cell during a burst to a more realistic value, however, without trying to catch the finer details of burst firing. Finally, our first model overestimated the membrane conductance at hyperpolarized membrane potentials, but underestimated it for depolarized membrane potentials, suggesting that the (voltage-dependent) input resistance of the model was too high at hyperpolarized membrane potentials, but too low at depolarized membrane potentials. We tuned the resistance at depolarized membrane potentials by increasing the overall input resistance ${\bar{g}}_{\text{pas}}$, while at the same time decreasing the capacitance *C*_*m*_, to fine tune the membrane time constant to the experimental value. To this aim we used the −50 mV initial membrane potential traces as a comparison, since these are least affected by the h-current (see below). To selectively increase the conductance at hyperpolarized membrane potentials, we added an h-current (maximal conductance ${\bar{g}}_{h}$, as used in [32, 33]) to all compartments, and adjusted its conductivity and mean input current amplitude to match the mean and the standard deviation of the membrane potential in the initial experimental traces at −80 mV. In this way the mean and the standard deviation of the membrane potential traces generated by the model were quite comparable to the experimental traces. This resulted in the following adjustments of/ additions to the original model:

$$\begin{array}{c} \begin{array}{cc} C_{m} & =0.75\cdot 0.9\cdot 0.88=0.59\mu F/{\text{cm}}^{2}\\ {\bar{g}}_{\text{pas}} & =0.75\cdot 37.9=28.4\mu S/{\text{cm}}^{2}\\ {\bar{g}}_{T} & =1590\mu S/{\text{cm}}^{2}\\ {\bar{p}}_{L} & =50\mu m/s\\ {\bar{g}}_{KC} & =1000\mu S/{\text{cm}}^{2}\\ {\bar{g}}_{h} & =130\mu S/{\text{cm}}^{2}. \end{array} \end{array}$$ (1)

#### Comparing model and experimental data

**Fig 9:**
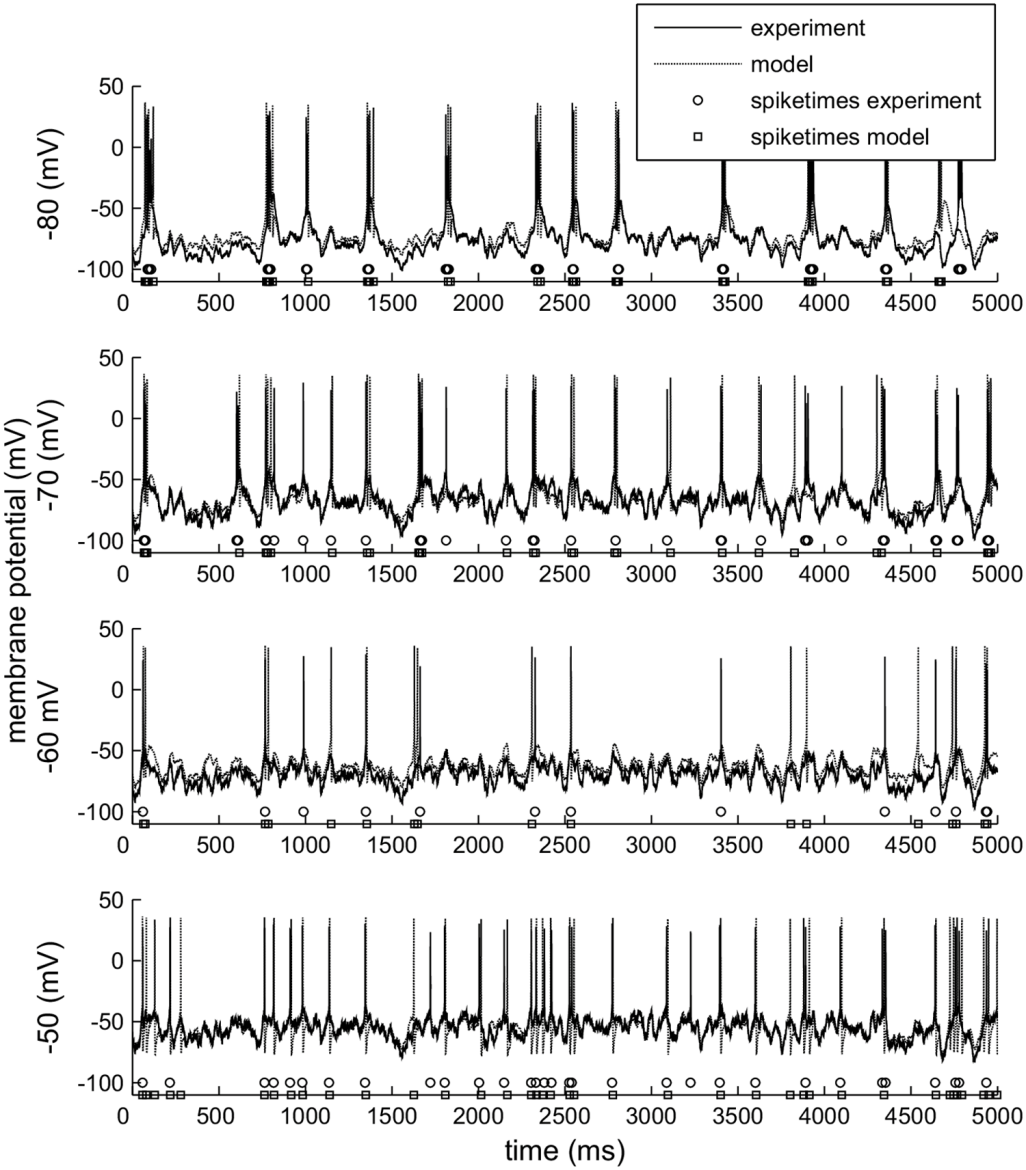
Comparing experimental and model spike trains. The same computer generated frozen noise was injected into the TCR cell (solid line) and in the fine tuned model cell (dotted line). This generated comparable spike trains consisting of isolated spikes and burst in a state dependent way (rows represent the four membrane states. Open circles indicate the timepoints at which the TCR cell fired, while squares mark the moments where the model cell fired, either a spike or a burst.

**Fig 10:**
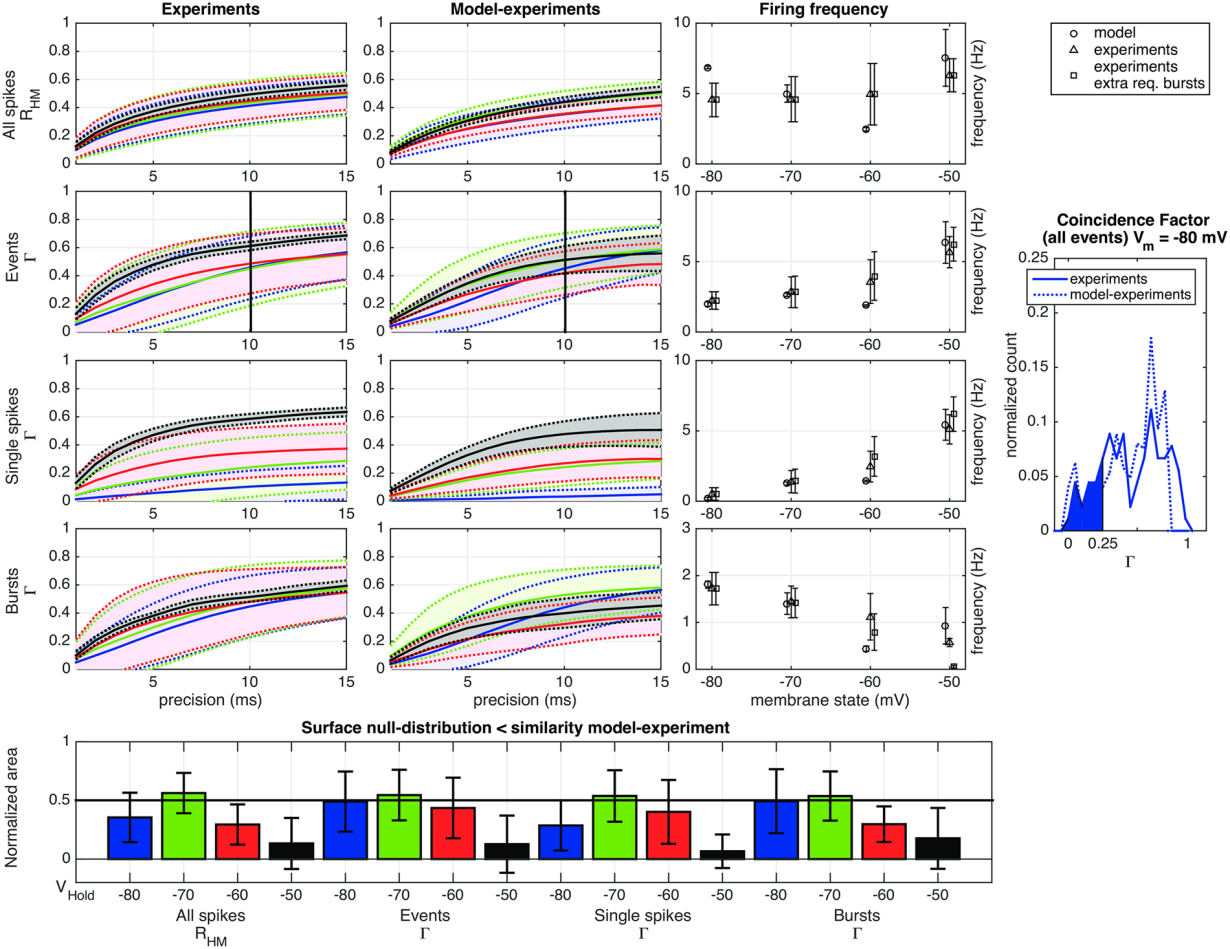
Comparing model and experimental spike trains. Left two columns: reliability (coincidence factor Γ or Hunter and Milton measure *R*_*HM*_) between experimental traces as a function of precision at different membrane states (blue: −80 mV, green: −70 mV, red: −60 mV, black: −50 mV). The shaded areas and dotted lines denote the standard deviations. The four rows represent the different types of activity: all spikes, all events (isolated spikess and bursts), isolated spikes and finally bursts. Second column: reliability between the model traces and the experimental traces. Third column: event frequencies for the model, the experiments without the extra requirement for bursts, and with the requirement (details in Materials and methods). Right panel: distribution of the reliability values among experiments (solid line) and between model and experiments (dashed line) of bursts at a precision of 10 ms (denoted by lines at this precision, see also S1 Fig). The (dis)similarity between between experimental spike trains on the one hand and the model and the experimental spike trains on the other, was established as follows: we established the null-distribution of experimental reliability values. Next, we calculated the surface of this null-distribution up to each model-experiment reliability value (shaded area). The bottom panel gives the values of these surfaces. If experimental and model spike trains were drawn from the same distribution, the expectation value of the mean surface would be 0.5, indicated by the horizontal line. Error bars indicate standard deviations.

Fig 9 illustrates the voltage traces (dotted lines) with spikes (squares) generated by the model and compares them with the experimental results (solid lines and open circles) using identical frozen noise in four predefined membrane potential states (rows 1 to 4). The details of the statistical comparison of the two experiments are further explained in Fig 10, where the third column gives the occurence frequencies for bursts (row 4), isolated spikes (row 3), events (row 2: isolated spikes and bursts) and all spikes (row 1: whether isolated or in bursts) for the model (squares) and the experimental recordings (triangles). Fig 10 also shows that at a membrane state of −80 mV, the majority of events were classified as bursts, whereas at a membrane state of −50 mV the neuron is in a spiking regime where most events consist of isolated single spikes, as discussed before in section ‘Burstiness’. In the experiments, the mean frequency of all spikes (top panel) was quite constant upon depolarization, whereas in the model it decreased when the membrane was depolarized from −80 mV to −60 mV, but then increased again on further depolarization to −50 mV. This is mainly due to the larger number of spikes per burst in the model, even after we implemented the calcium-activated potassium current and is confirmed by the fact that the isolated spike as well as the burst frequencies are quite similar in model and experiment (row 3 and 4). Because we were in this study not focussed on the fine details of the burst firing pattern, this deviation will not affect our conclusions and we did not try to further fine-tune the burst spiking pattern. Two other differences between model and experiment are 1) the smaller variance in the frequency between trials in the model and 2) the lower activity of the model at −60 mV. We can not completely exclude that the slightly larger variance in the experiments is partially caused by some form of slow adaptation that violates the assumption of stationarity, as has been discussed in a different paper [34].

**Fig 11:**
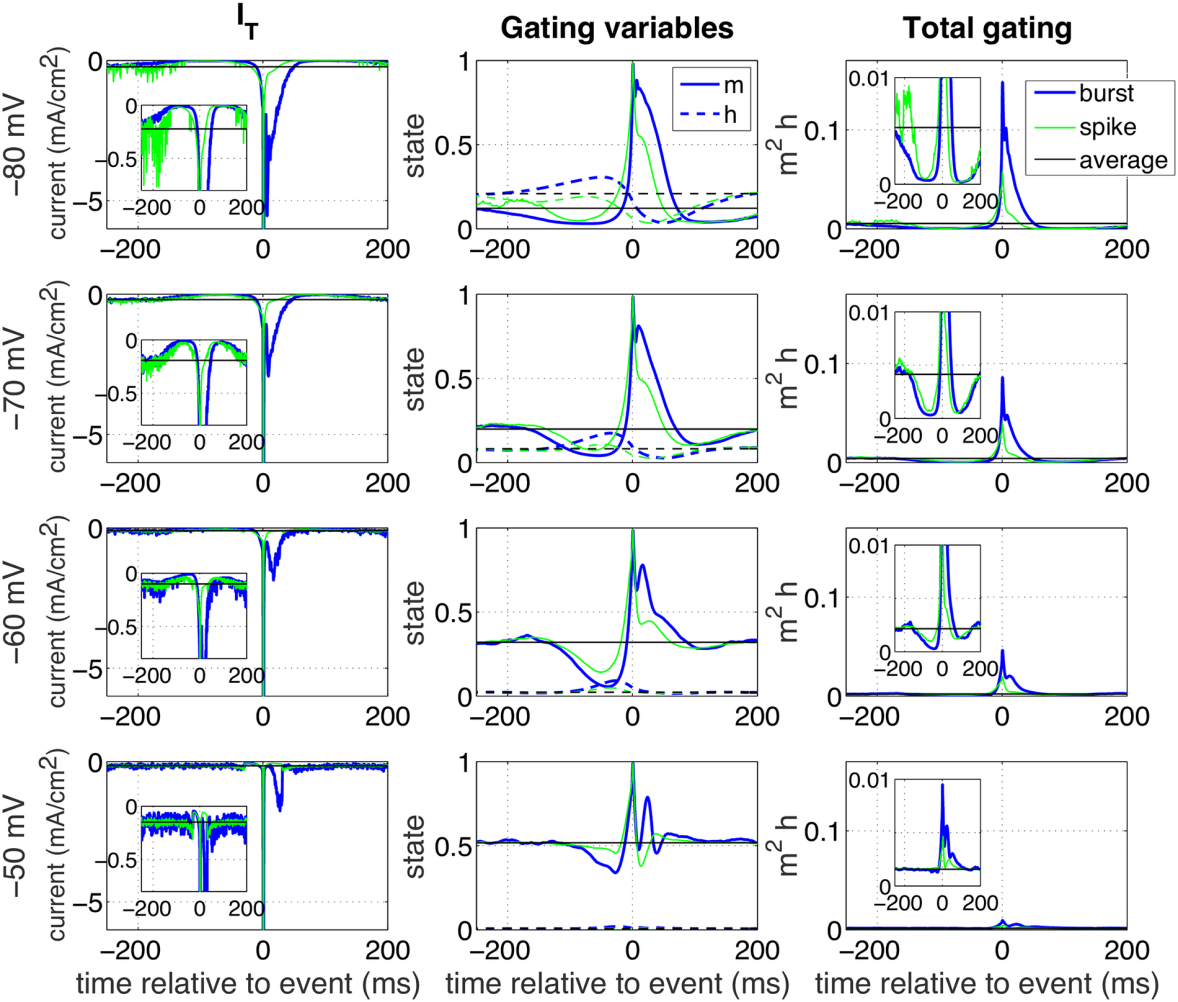
Event-triggered T-type calcium current. Event-triggered T-type calcium currents were calculated for the model (first column) comparable to the experimental ETAs in Fig 6; t = 0 indicates the peak of the (first) spike. Rows indicate the four different membrane states; blue traces indicate the current when a burst was induced, while green traces depict the situation for an isolated spike. The horizontal black line gives the mean value in the steady state condition. The inset zooms in around the time of initiation. The second column depicts the gating variables *m* (solid) and *h* (dashed) that underly the current using the same color code. The third column illustrates the total gating (the product *m*^2^*h*) that underlies the current.

The reliability of the experimental traces, and the quantitive comparison between the model and experimental traces were further analyzed using the coincidence factor Γ (Fig 10, first column) and Hunter and Milton’s measure *R*_*HM*_ (Fig 10, second column). The distribution of the reliability between experimental values at a precision of 10 ms was used to construct a null-distribution (Fig 10, right panel). For each reliability-value between the model and experiment (i.e. a comparison between a spike train of the model and an experimental spike train), the surface of the null-distribution up to this value was calculated. The mean and the standard deviation of these surface values are shown in the bottom row of Fig 10. If the experimental and the model spike trains were drawn from the same distribution, the expectation value of the surface values would be 0.5 (black line). If the model spike trains are more dissimilar from the experimental spike trains than the variability between experimental spike trains, the mean of the surface values would lie below 0.5 and vice versa. From Fig 10 we conclude that the model describes the experiments quite well at a holding potential of −70 mV (green bars). At −80 mV, the model performs better for bursts than for isolated spiks (blue bars), but since spikes are rare, the power of that comparison is low. At −60 mV (red bars), when there are less bursts and more isolated spikes, the model describes the isolated spikes well. At a holding potential of −50 mV the model is a less good description of the experimental spike trains. The main goal of our model was to asses the role of the h-current and the T-current in burst and spike generation. At a holding potential of −50 mV *I*_*T*_ is almost completely inactivated and *I*_*h*_ is hardly activated (see Fig 11). Improving the performance at −50 mV by implementing a substantial set of additional ionic currents (and so strongly increasing the number of parameters in the model) was considered beyond the scope of this study. We conclude that for the relevant membrane voltage range the model is a good representation of the real neurons in our experiments.

Once the spike times (Fig 9) generated by the injection of identical frozen noise are well comparable between the model and the experiments, it is not surprising that the analytical tools that quantify the spike trains of the model lead to the same conclusions as they did in the experiments. Although we performed this basic quantitative analysis, we will not reiterate these results here and suffice with this conclusion. We concentrate on the aspects in the model that cannot easily be revealed experimentally.

#### Event-triggered T-type calcium current

The experimental results suggest that for the low membrane states the depolarization induced by the T-type calcium current is not only needed to initiate bursts, but also to initiate isolated spikes. This was concluded from the ETAs (Fig 6): in hyperpolarized states they are similar for spikes and bursts, and therefore particularly slow for isolated spikes. Moreover, the coherence (Fig 4) shows that at hyperpolarized states single spikes do not show the broadband phase locking at the peak of an oscillation (phase = 0 rad), but only low-frequency phase locking, with a later phase for higher frequencies. The model allows us to directly test this hypothesis. To this aim we calculated the ‘event-triggered T-current’ (Fig 11, left column). The T-type calcium current was implemented with three gating variables, two identical activation gates *m* and one inactivation gate *h*. We calculated the ETA for these activation gates (Fig 11, second column) and also for their product *m*^2^*h* (denoted by ‘total gating’, Fig 11, third column) for all four membrane states (rows).

The biggest difference between bursts (blue traces) and spikes (green traces) can be seen after the (first) spike: The T-current (first column) and the total gating (third column) are higher for bursts (blue traces) than for spikes (green traces). The inactivation gate (middle column, dashed lines) is further opened before bursts than before spikes. As expected, there is a reasonable T-type calcium current activation before single spikes in hyperpolarized states, which could explain the high burst-like behaviour of single spikes found in the experiments for the hyperpolarized states. Two more conclusions were drawn from our simulations: firstly, even though it is hardly visible in the total activation (or the total current), the graphs of the individual gating variables (middle column, note the difference in scale on the horizontal axis) show that the deviations from the mean for the individual gating variables start very long, often hundreds of milliseconds, before the spike or burst in the bursting regime. This explains why, in this regime, the intervals for these events need to be so large in order to consider them independent. Secondly, at a membrane state of −80 mV the activation of the T-type calcium current about 10 ms before the event is higher for a single spike than for a burst (insets). So apparently, the difference between a burst and a spike is not only the result of the total activation level of the T-type calcium current, but also the dynamic activation profile plays an important role as: for an isolated spike the total activation is slower, so the activation gate can open a bit more, before it is overpowered by the inactivation gate.

#### Contribution of h-type current

**Fig 12:**
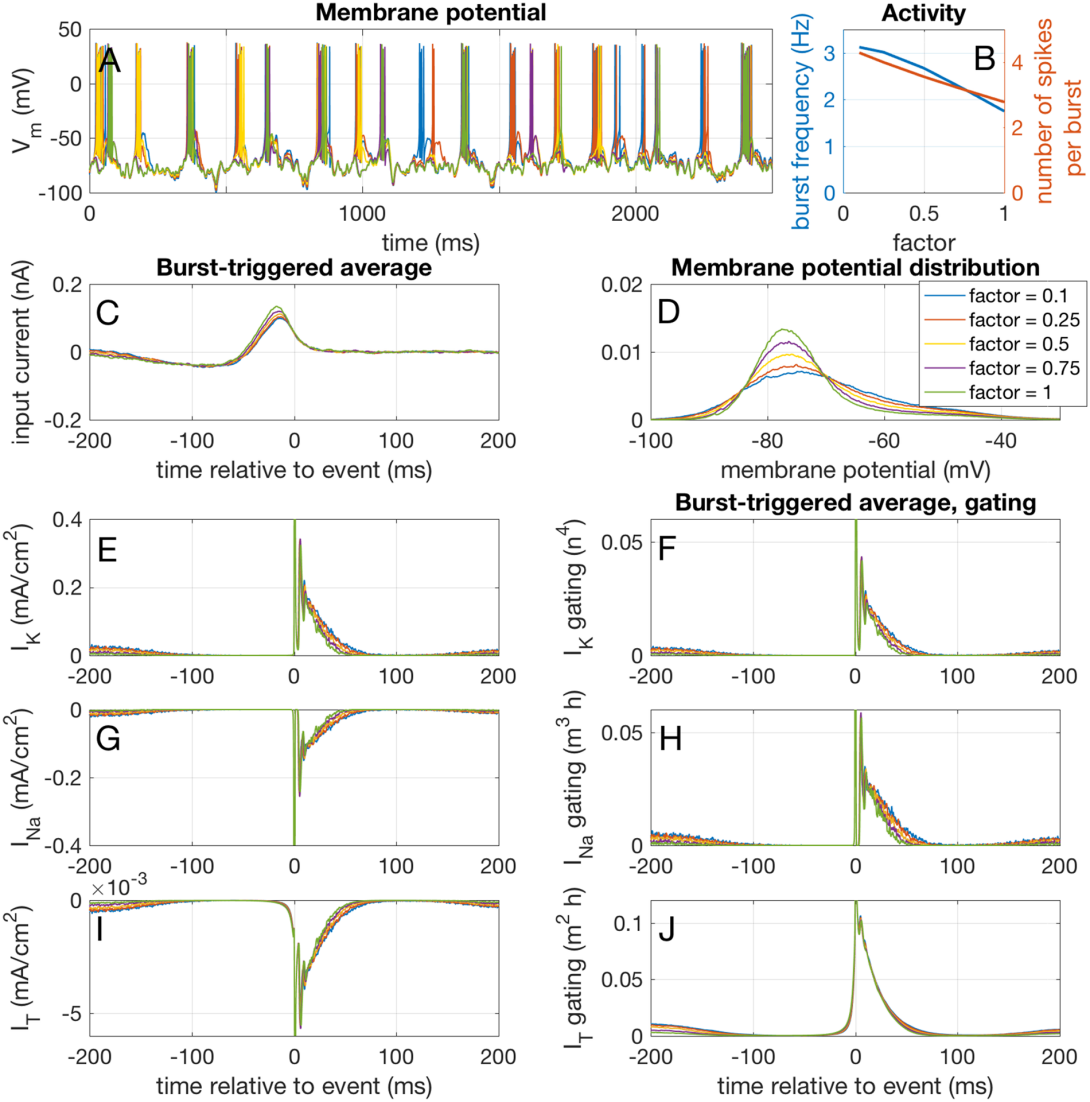
Effects of reducing the h-type current. The maximum conductance of the h-type current ${\bar{g}}_{h}$ and the mean of the input current (-700 pA) were reduced with the same factor *f* in the bursting regime. This resulted in a reduction of both the burst rate and the number of spikes per burst, as can be seen from the example traces in A and the event frequencies in B. Reducing the h-type current and mean input current resulted in a reduction of the width of the membrane potential distribution, while keeping the mean membrane potential at the same level (D). A stronger deviation from the mean input current was needed to trigger a burst (C). The burst-triggered potassium (E), sodium (G) and T-type (I) currents and gating (F, H and J respectively) show that this reduction in the width of the membrane potential distribution affects all three currents.

In order to mimic the membrane voltage state dependency of the firing we had to fine-tune a second ionic current in the model that is active in the subthreshold voltage range. The hyperpolarization activated non-inactivating h-type current (reversal potential: -40 mV) is well suited for this purpose and present in TCR cells [5, 6, 17, 18]. The amplitude of the current in the model was adjusted to tune the voltage dependent firing rates (Fig 10). The contribution of *I*_*h*_ to the bursting regime was investigated by reducing the maximum conductance of the h-type current ${\bar{g}}_{h}$ while at the same time reducing the mean of the input current (-700 pA) with the same factor *f* (so *f* = 1 corresponds to the maximum injected current and *I*_*h*_, *f* = 0.1 to the minimum). By reducing both these currents, the mean membrane potential was kept constant, so the neuron stayed in the bursting regime (Fig 12D). Reducing both the mean injected current and *I*_*h*_, gave us the opportunity to study the effect of a dynamic (*I*_*h*_) depolarizing current, as opposed to a constant current. In Fig 12B it is shown that *I*_*h*_ reduces both the burst rate (blue line) and the number of spikes per burst (red line). Indeed, a stronger deviation from the mean input current was needed to evoke a burst (Fig 12C), so it was ‘more difficult’ to evoke a burst. *I*_*h*_ reduces the width of the membrane potential distribution (Fig 12D). Therefore, we hypothesized that this reduced the deinactivation of excitatory currents with an inactivation particle, such as *I*_*T*_ and *I*_Na_. We investigated this using the ETAs in a similar way as described above for *I*_*T*_. Indeed, *I*_*h*_ has an effect on the deinactivation of both the sodium current (Fig 12G and 12H) and the T-type calcium current (Fig 12I and 12J), reducing their net activation and current. It also reduced the net potassium current (Fig 12E and 12F), but apparently not enough for an excitatory effect. In conclusion, using the voltage dependent *I*_*h*_ instead of an injected current of constant amplitude reduces the width of the membrane potential distribution, having effects on many other membrance currents, inactivating ones in particular. This effect will be strong in the bursting regime, where *I*_*h*_ activates and deactivates most strongly.

#### The high-conductance state

**Fig 13:**
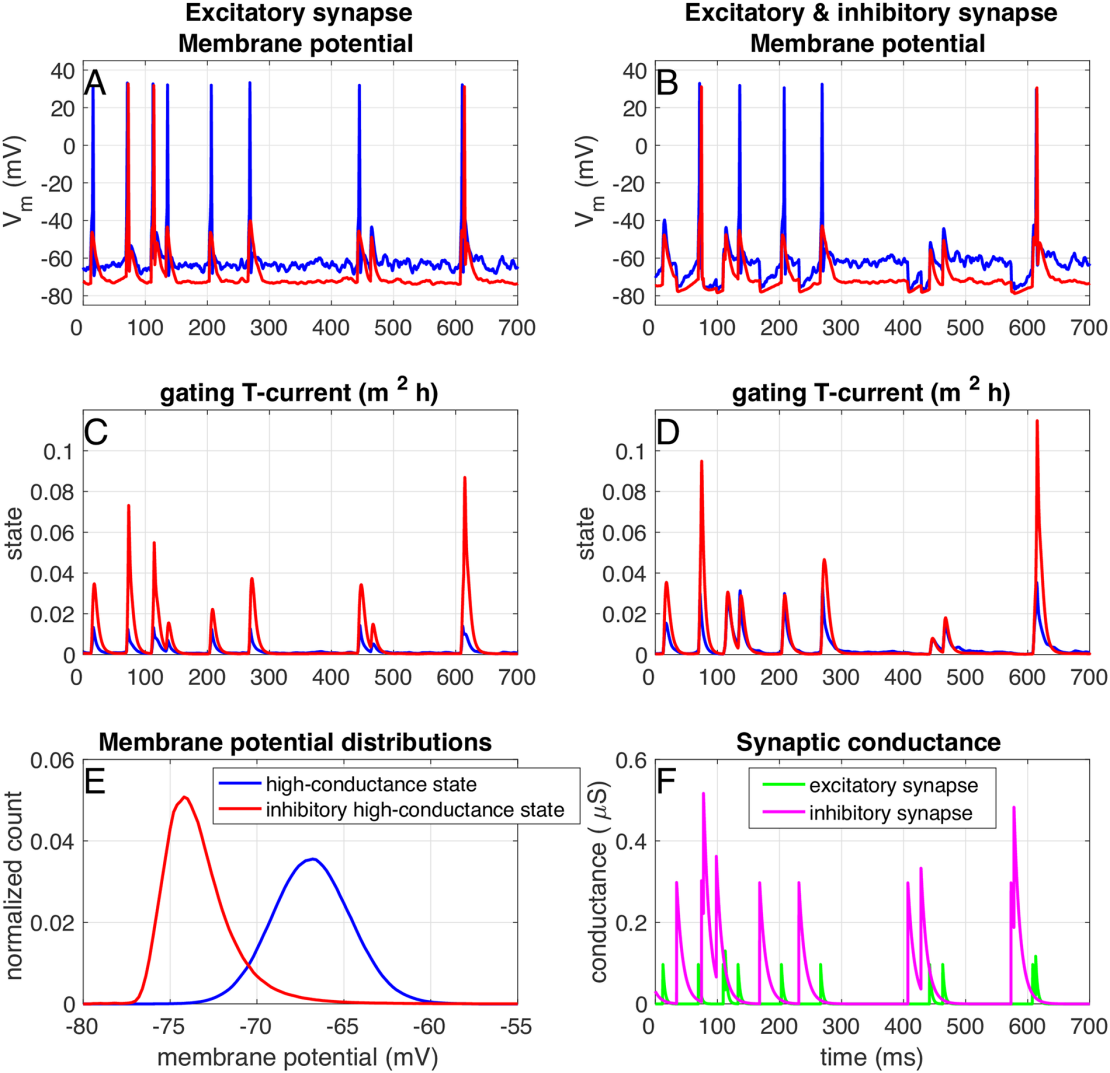
The high-conductance state. In a ‘standard’ high-conductance state [28] (blue traces), the membrane potential fluctuates around a depolarized value (E). The neuron responds to excitatory input with single spikes (A), because the T-type calcium current is inactivated (C). Inhibitory input that is synchronized with (arrives just before) the excitatory input (F) can deinactivate the T-type calcium current, so that it is activated upon the arrival of excitatory input (B and D). In a ‘inhibitory’ high-conductance state (red traces) the membrane potential is not depolarized (E). Due to this lack of depolarization, the inactivation is removed from T-type calcium current and the T-current can activate for excitatory input only (C).

The frozen noise input offered an excellent opportunity to determine to what feautures in the input signal a neuron responds and what the optimal stimuli are to induce either isolated spikes or bursts. It mimics the fluctuating membrane voltage, that under normal conditions occurs in most cells, but it does not mimic the enlarged conductance typically found in the so-called high-conductance state that results from the continuous *in-vivo* bombardment with synaptic signals [28]. Therefore, we also implemented a more realistic version of the model that included a high-conductance state following [35] (see Material and methods): we implemented two passive synaptic conductances, one excitatory and one inhibitory, with fluctuating conductance. The standard deviation of the fluctuations of the subthreshold membrane potential was around 2 mV (Fig 13E, blue line) as was reported before [28]. A stimulus, a second excitatory exponential synapse (*τ* = 3 ms, conductance 100 nS) was added to the proximal dendrite, and activated by a Poisson spike train with a mean inter-spike interval of 100 ms (‘Randomstream’ template, [36, 37], modeldB entry 83319). With these settings the model neuron responded to 85,7% of the inputs. The burst frequency was about 20,0% (number of bursts divided by the number of events, see Fig 13A, blue line). However, these ‘burst events’ are a mix of intrisic burst (generated by the T-type calcium current), and short inter-spike intervals caused by short interval stimuli (fraction of ‘input bursts’: 26,8%). The T-type calcium current was inactivated due to the depolarized membrane potential (Fig 13C, blue line), so indeed most of the ‘bursts’ are responses to stimuli with a short inter-stimulus interval. To activate the T-type calcium current, hyperpolarization of the membrane potential is needed to remove inactivation. We hypothesized that this could be accomplished most effectively in multiple ways: either by 1) asynchronous ‘surround’ inhibition, or by 2) inhibition that is well-timed with the excitatory input. The first scenario was modelled by changing the ‘standard’ high-conductance state to an ‘inhibitory’ high-conductance state: all membrane potential fluctuations were now caused by an inhibitory synapse, that matched the total conductance in the ‘standard’ high-conductance state (see Materials and methods). As can be seen in Fig 13A and 13C (red lines), in such an ‘inhibitory’ high conductance state the T-type calcium current was activated, even though still not many bursts were fired (response fraction: 24%, burst fraction: 8,1%). The second scenario was implemented by adding an inhibitory synapse (*τ* = 10 ms, conductance 300 nS) to the proximal dendrite. This inhibitory synapse was activated by the same Poisson spike train as the excitatory synapse, but shifted 35 ms towards earlier times (Fig 13F). In this case too the T-type calcium current was activated (response fraction: 57,0%, burst fraction: 1,0%, Fig 13D, blue line). Finally, when both scenarios are combined (response fraction: 13,3%, burst fraction: 2,2%, Fig 13D, red line), the T-type calcium current is strongly activated for each input event, but the neuron does not respond much. Moreover, we note that both the conductance and time-shift of this inhibitory synapse need to be precisely fine-tuned: if the inhibitory conductance is too high, or the inhibitory event arrives too late, the excitatory event is effectively blocked by the inhibitory synapse. If the inhibitory conductance is too low or the event arrives too early, the T-type calcium current is not deinactivated enough to activate upon arrival of the excitatory synapse. We conclude that under *in vivo*-like conditions, inhibition is needed for the T-type calcium current to be activated. This can either asynchronous ‘surround’ inhibition, or inhibition that is well-timed with the arrival of excitatory events.

## Discussion

The main purpose of this study was to reveal the input code to which a rat thalamocortical relay (TCR) neuron responds and which features in that signal determine whether an isolated spike or a burst will be generated. Information decoding in the TCR cell is mostly done by voltage dependent ionic currents and this directly implies that the actual mean membrane voltage at which the cell operates (which we have denoted the ‘membrane state’ in this study) is highly relevant. Voltage dependence is specific for each participating ionic channel, which has as a consequence that even for the same molecular expression level the functional ionic current composition changes with membrane state; such switches can be very fast and are fully reversible.

In this study, we used a combined experimental-modelling approach. Experimentally, we used (computer generated) frozen noise with a defined amplitude superimposed on a DC level to evoke a well reproducible spike train in the TCR cell, so that we could use the exact same signal in the model cell. A slow feedback system guaranteed the right starting voltage in the experiments; this means that small drifts during the recording were not compensated in order not to introduce artificial low frequency components. The time-invariant good match between experimental and model trains, suggests that such drifts do not play a critical role. Experimentally, we first analysed the spike and burst triggering features of the input pattern using the Event-Triggered Average (ETA) and Event-Triggered Covariance (ETC) [23, 25, 26] and we calculated the information that is carried by the various elements of the input triggered response as a function of the membrane state or regime (i.e. a low holding potential results in a bursting regime, an intermediate holding potential in a mixed regime or a high holding potentialin a spiking regime).

We found that in a mixed regime, spikes transfer different information than bursts: spikes phase-lock to and transfer information at higher frequencies than bursts. Spikes are also more selective for fluctuations than bursts. Finally, a single spike contains less information than a burst, but especially in the spiking regime there are many more of them, so they do carry the bulk of the information in the spike train. Bursts on the other hand are highly informative, but in the spiking regime rare. They can only phase-lock to and transmit information at low frequencies, and are especially in the hyperpolarized bursting regime a response to slow integration rather than fast fluctuations. This is in agreement with Lesica and Stanley [38], who claim that because of these low-frequency properties of bursts, white noise causes less bursts than more natural or pink noise, in which frequencies decay according to a power-law. In contrast to our conclusions, Reinagel et al. [8] claim that thalamic bursts and spikes code for similar information, but since they used visual stimuli with a cutoff frequency of about 16 Hz, they could not see the different frequency sensitivity we found here, a limitation they mention in their discussion.

On depolarization the neuron goes from the bursting to the spiking regime, in which it responds earlier in time, more precisely (an effect of the slope of the input-output frequency curve [34]), more to fast fluctuations and less to slow integration. Due to this transition it is capable of responding to higher frequencies: it can phase-lock to them and transfer information at higher frequencies. Such frequency dependence of information transfer by spikes and bursts has been investigated before, although with less general inputs [39–41]. This frequency effect can also be seen in the properties of the subthreshold membrane potential: on depolarization the impedance of the subthreshold membrane increases at higher frequencies. However, even in the spiking regime the information transfer remains bound to relatively low frequencies: both the impedance and the information density strongly decay for frequencies higher than about 50 Hz. Such depolarisation can result *in vivo* from, for instance, cortical input from L6: Mease et al. [42] showed that L6 stimulation resulted in reduced thalamic adaptation, which in turn gated the relay of high-frequency sensory inputs. Indeed, it has been shown that whether TCR cells are in a bursting or a spiking ‘state’ is an effect of the visual stimulus (history), so that the ‘state’ of the cell encodes visual information [43]. The coherence showed that in the low membrane potential bursting regime, spikes behave like bursts in that they do not phase-lock to the peak of the oscillation, but show a phase delay for higher frequencies. The spike-triggered membrane potential and current show in this regime large negative deflections, all suggesting that in this regime single spikes are more ‘one-spike bursts’ than true spikes, and that they need the activation of subthreshold ionic currents to be initiated.

Our computational model, fine-tuned to represent the experimentally observed spike trains in response to the identical frozen noise stimulus, allowed to infer the role of the most important subthreshold ionic currents in spikes and bursts (*I*_*T*_ and *I*_*h*_), information that can experimentally hardly be obtained as the needed manipulations would catastrophically interfere with cell behavior. Because the main goal of our model was to asses the role of the h-current and the T-current in burst and spike generation we accepted a less then perfect fit for the membrane state −50 mV, where the h-current is not activated and the T-current is inactivated. Improving the performance at −50 mV by implementing a substantial set of additional ionic currents (and so increasing the number of parameters in the model) was considered beyond the scope of this study. We also did not try to reproduce the finer details of the experimental bursting patterns of the TCR neurons as this would certainly require a more advanced calcium homeostasis and a wider repertoire of *Ca*^2+^ dependent *K*^+^ currents. Thus bursts were classified only as bursts without further analysis of the detailed bursting pattern. We conclude that for the relevant membrane voltage range the model is a good representation of TCR activity as observed in our experiments.

Since the spike times (Figs 9 and 10) generated by the injection of identical frozen noise are well comparable between model and experiments, it is not surprising that the analytical tools that quantified the experimental spike trains yield similar results for the model. Therefore, we focus on the model results that cannot be revealed experimentally: the event-triggering T-current and h-current. At strongly hyperpolarized membrane states, there is a considerable T-current activation, even for single spikes. The ETAs calculated for the activation and inactivation gate of this current indicate that the deviation from the mean, for spikes as well as for bursts, already starts hundreds of milliseconds before the event, which explains the long interaction times in the bursting regime. The activated T-type calcium current about 10 ms before the event is larger for a spike than for a burst. This might sound counterintuitive, but the higher membrane potential (Fig 11) causes the inactivation gate to be more closed, but the activation gate to be more open. So the total activation has a different profile for spikes than for bursts: even though the T-current has a higher amplitude about 50 ms before a spike as compared to before a burst, the dynamics allow the gating variables to open more during a burst. Apparently the initial slope plays a more important role than the initial amplitude. The h-current has a similar (to the T-current) state dependent contribution to the event generation, be it with a simpler dynamics. It contributes maximally at low membrane voltages, where its driving force (reversal potential around -40 mV) results in a depolarization of the membrane. Around −50 mV there is very little *I*_*h*_ activation. The dynamic interaction between *I*_*h*_ and *I*_*T*_ has theoretically been analysed in detail before. Several researchers showed that the *I*_*h*_ and *I*_*T*_ pair can cause oscillations [16–19, 44]. Here, we extended this knowledge by showing that in the bursting regime, the dynamic *I*_*h*_ reduces the width of the membrane potential distribution, which results in a reduction of both the burst frequency and the number of spikes in a burst through interacting with several ionic currents. This effect is strongest in the bursting regime, because *I*_*h*_ is both most active and has the largest gain in this membrane potential region (half-activation value around −75 mV). So *I*_*h*_ effectively ‘clamps’ the membrane potential in the bursting regime.

In vivo, neurons receive a constant bombardment of synaptic inputs, resulting in the so called ‘high-conductance state’ [28]. In the in vitro current clamp experiments, the fluctuating input current did mimic the fluctuating membrane voltage, but it does not originate from an enlarged conductance. Therefore, we repeated the model simulations in an *in-vivo*-like high-conductance state. Our conclusion that the burst frequency is low in a ‘standard’ high-conductance state, because *I*_*T*_ is inactivated, confirms the findings of Swadlow and Gusev [11], who measured the response of the neocortex to thalamic bursts in awake rabbits and found that the neocortex is powerfully activated by bursts. Moreover, even under anaesthesia, bursts frequencies are low, especially when white noise stimuli are used [43]. ‘Natural’ stimuli induce a higher burst rate.

Paradoxically, the synaptic bombardment of the high-conductance state increases the burst rate by decreasing the difference between the spiking and the bursting regime, effectively mixing them [45]. However, they compared the number of bursts in a ‘standard’ high conductance state to a quiescent state (without background input) at around -63 mV. We did not simulate such a ‘quiescent’ state, but we do not expect the T-type calcium current to be active in such a quiescent state, due to the half-values of its activation and inactivation gates (-56 mV and -80 mV respectively). These authors find that under noisy conditions, about 20 to 30 percent of their synaptic stimuli result in bursts, whereas in our high-conductance state simulations the burst rates are much lower. This discrepancy could have several causes: firstly, Wolfart et al. find the high burst rate in response to a regular stimulation at a frequency of 5 Hz; at higher stimulation frequencies the burstiness was much lower. Our stimulation had a higher frequency (10 Hz) and was Poissonian, both resulting in a larger representation of short inter-stimulus intervals. Most likely, this limits bursting, because the T-type calcium current needs pre-stimulus hyperpolarization to de-inactivate, which will not happen at short inter-event intervals. Indeed, we confirm here that bursts have a resonant frequency of about 5 Hz [46]. Secondly, we used a different inter-spike interval constraint for burst detection (30 ms) than Wolfart et al. (‘a large ISI (≥ 50 ms) followed by one or more small ISIs (≤ 6 ms)’). We show that the most effective way of activating the T-type calcium current in the high-conductance state, is having either a strong inhibitory surround (‘inhibitory’ high-conductance state), or inhibition that is fine-tuned with the excitatory input in both timing and size, for instance in a feed-forward structure. Wang et al. [47] have observed that in cat visual thalamus *in vivo*, there is strong inhibition before bursts that originates from the central subregion. Even though it is difficult to investigate T-type calcium current activation *in vivo*, several labs have measured thalamic spikes and bursts *in vivo*, in particular in cat visual thalamus [47–49]. They found that both single spikes and bursts need ‘suppression’ before event onset and burst need more ‘suppression’ than single spikes, which is in line with our results. Moreover, *in vivo* bursts are more reliable than single spikes [48], something we have also concluded before [34]. This suggests that the bahaviour of our TCR cells *in vitro* is comparable to the *in vivo* situation.

The thalamus is thought to have a ‘relay’-function in the information stream from sensory modalities to the cortex: it gates and modulates the information flow to the cortex [50]. How information is ‘relayed’, what information is stopped or allowed to pass to the cortex and how this information is modulated, is still largely unknown. In particular, why the relay cells in the thalamus use bursts in their communication is still an open question. The finding that bursts in *in-vivo*-like states are rare but highly informative is in agreement with the main functional hypotheses about the role of thalamic bursts: a ‘wake-up call’ [9], ‘feature detection’ [51] or ‘searchlight’ role [52], as opposed to a ‘stimulus estimation’ role for single spikes [38, 51, 53]. In these hypotheses, the brain uses thalamic bursts to focus an ‘attentional searchlight’, i.e. thalamic bursts are signals that ‘something has changed’. Tonic spikes are then used to convey what is going on, because they are able to convey information at a broader frequency range. Also, bursts are thought to initiate up-states [54], which fits with the same hypotheses: a burst at the start of an ‘up-state’ signals that ‘*something* is going on’, and the following tonic spikes in the up-state convey ‘*what* it is that is going on’. An additional functional relevance of a burst is that it allows a more secure signal propagation in the sense that the likelihood of a failure is strongly reduced. Our conclusion that well-timed inhibition can either prevent or initiate a burst, sheds interesting light on the ‘relay’-function of the thalamus. Since inhibition to TCR cells comes from thalamic reticular cells, that in their turn receive their input from TCR cells and primary sensory cortices [55], this means that what information is transferred by TCR cells and whether this is done by spikes or bursts is under strong feedback control that could make the thalamus selective for new or unexpected information, while suppressing expected information.

The features that trigger a bursts depend on the biophysical properties of the burst-generating neuron. In a minimal computational model of pyramidal neurons [56], in which a burst is generated by an interplay of a slowly depolarizing dendrite and a spiking soma, it has been shown that bursts occur during positive slopes if the input signal, and that the number of spikes per burst correlates with the steepness of the slope [57, 58]. As TCR cells use a different mechanism for burst-firing (T-type calcium current instead of a ‘ping-pong’ interplay between soma and dendrite), TCR cells cpuld well be sensitive to different features in the input than pyramidal cells: in TCR cells single spikes and bursts are caused by different features, while they are caused by similar features in pyramidal cells. Nevertheless, bursts are very robust in both cell types [34]. Because of the dependence on the burst-generating mechanism, conclusions about the functional role of bursts will most likely vary strongly between brain regions and species: in weakly electric fish bursts are thought to have a ‘feature detection’ role [51, 59], like in thalamus. In mouse hippocampus it was shown that the role of bursts in the coding of contextual fear memories is very different from the role of bursts in the prefrontal cortex in the same animals [60, 61]. Neurons in the auditory system of grasshoppers however, transfer a significant amount of information via the number of spikes in a burst (depending on the type of bifurcation leading to a burst) [62, 63]. A similar code was found in *in vivo* experiments in cat LGN [49, 64] as well as in single-compartment models of TCR neurons [65]: the authors suggest an ‘*n*-spike burst code’ for thalamic bursts, in which the number of spikes in a burst signals different stimulus feature intensities. Recently Mease et al. [66] showed that TCR cells use a multiplexed code, in which there is information in burst size as well as burst onset time and precise spike timing within bursts. We have shown here experimentally that there is a clear difference between encoded features for a single spike (*n* = 1) or a burst (*n* > 1), and that the encoded features span tens to hundreds of milliseconds before and after burst onset, in agreement with such a ‘*n*-spike burst code’. However, a spike train with only isolated spikes and first spikes of bursts carried about the same amount of information about the input as a spike train where all spikes were included, suggesting that the ‘follower’ spikes in bursts do not carry much information, as shown previously [67]. This might be an effect of the low-pass filter (exponential filter with *τ* = 10 ms) we used. The fast component in the burst-ETA for the more broadband (*τ* = 1 ms) input, suggests that there is information that is transferred at higher frequency bands for bursts, possibly in the spike timing within a burst. However, given the low burst frequency for the broadband input, we could not properly differentiate this in our analysis. So whether the number of spikes in a burst carries significant information, probably depends on the experimental setup and the used stimuli. Indeed, as Gaudry and Reinagel [64] noticed before, in many recordings bursts are highly stereotyped and bursts don’t differ enough from one another to analyse the information carried in within burst information. However, in recordings where the differences between bursts are strong, these differences have been shown to carry (additional) information.

We conclude that in rat thalamus, TCR cells use bursts to represent other features than single spikes: bursts phase-lock to and transfer information at lower frequencies and they are less selective for fluctuations. If the neuron is depolarized, it goes smoothly from a bursting to a spiking regime, in which it is more sensitive to high-frequency fluctuations.

## Materials and methods

### Experiments

#### Ethics statement

Experiments were approved by the animal welfare committee of the University of Amsterdam.

#### Slice electrophysiology

Electrophysiological experiments were performed using brain slices from wistar rats (postnatal days 12-16, Harlan, Zeist, Netherlands) anesthetized with isoflurane and killed by decapitation. 300 *μ*m-thick coronal slices containing thalamic nuclei were cut at the level of the hippocampus with a vibroslicer (Leica VT1000S, Wetzlar, Germany) in ice-cold slicing solution containing in (mM): Choline chloride (120), KCl (3.5), CaCl_2_ (0.5), MgSO_4_ (6), NaH_2_PO_4_ (1.25), NaHCO_3_ (25), glucose (25), continuously bubbled with 95%O_2_ − 5%CO_2_ (pH = 7.4). Slices were incubated at 32°C for 1 h in artificial cerebrospinal fluid (ACSF) containing (in mM): NaCl (120), KCl (3.5), CaCl_2_ (2.5), MgSO_4_ (1.3), NaH_2_PO_4_ (1.25), NaHCO_3_ (25), glucose (25), continuously bubbled with 95%O_2_ − 5%CO_2_ (pH = 7.4). During recording, slices were kept submerged at room temperature (20 − 22°C) and were continuously superfused with ACSF. Patch pipettes were pulled from boroscilicate glass and had a resistance of 2–3 MΩ when filled with internal solution containing (in mM): K-gluconate (130), KCl (10), EGTA (5), HEPES (10), Mg-ATP (4), Na-GTP (0.4) pH = 7.3. Current-clamp recordings were made using an EPC9 patch-clamp amplifier controlled by PULSE software (HEKA Electronic GmbH, Germany) and in-house data acquisition software running under MATLAB (MathWorks, Natick, MA, USA) using a NIDAQ USB-6259 interface (National Instruments, Austin, TX, USA). Signals were filtered at 5 – 10 kHz and sampled at 10-20 kHz. Membrane potentials were corrected for a 10 mV liquid junction potential.

Cells were identified based on region (dorso-ventral thalamus), morphology (several were filled with biocytin and reveled the typical TCR shape) and the presence of burst responses in a simple step protocol. The input resistance was 56 ± 6 MΩ (mean ± sem, *n* = 6), the resting membrane potential was −61 ± 4(*n* = 6) mV.

#### Noisy input trains

In current-clamp measurements the cell was injected with current that consisted of a DC component with superimposed noise: a computer generated (MATLAB) time series of Gaussian distributed random numbers filtered by an exponential filter. Two configurations were used: 1) the filter had a time constant of *τ* = 10 ms and a standard deviation of *σ* = 100 pA or 2) the filter had a time constant of *τ* = 1 ms and a standard deviation of *σ* = 200 pA. A slow feedback system controlled the background DC current to stabilize the membrane voltage at one of the specified values (−80 mV, −70 mV, −60 mV or −50 mV) before the actual recording started; after the start this DC current component was fixed. The same frozen (= an exactly reproduced computer generated) noise train was injected into the soma of the TCR neuron for every repetition of the experiment. The noise amplitude was fine-tuned so that the neurons fired with a mean spike frequency of 1.7 – 6.5 Hz, low enough to assume that the inter-spike intervals (150 – 500 ms) were of sufficient length to consider most events independent of each other. As soon as the current injection evoked spikes and bursts, the recorded mean membrane voltage was slightly different from the intended one. We could have kept the feedback system running, but we preferred this small constant bias over adding a difficult to control low-frequency component to the recording. Wherever relevant we have indicated the measured voltage in the figures; for reasons of clarity we will refer to them as membrane states of −80 mV, −70 mV, −60 mV and −50 mV. In most experiments we used at least three repetitions of 300 s duration at the four distinct membrane states. To compare regimes (bursting, spiking or mixed) and to have suffiecient events to calculate the ETC we also recorded traces of 900 s duration.

The model was activated by current traces identical to the ones used in the experiments. They were scaled to an amplitude that induced similar voltage fluctuations as observed in the experiments and their DC-component was adjusted so that the average subthreshold membrane potential was comparable to the one in the experiments.

#### Spike and burst detection

Action potentials were detected by level crossing (Fig 1) using a threshold that varied between −23 and 0 mV which also accommodated spikes with a low amplitude. Whether spikes were isolated single spikes or part of a burst was decided based on the inter-spike intervals (ISIs). In the presence of bursts the ISI histogram shows two peaks and the return map (graph that plots interval n against interval n+1) can be divided into four distinct regions (Fig 2) using a k-means algorithm and the silhouette value for statistics. Combining these forms of analysis provided a robust criterion to separate isolated spikes from bursts. In the experiments (but not for the model) an additional criterion was used: in a burst the membrane potential between the first and second spike should stay at least 10 mV above the mean membrane potential: min((*V*(*t*_1_),*V*(*t*_2_)) > 〈*V*_*m*_〉 + 10; this ensured that bursts consisted of spikes riding on top of a low-threshold calcium spike.

Covariance analysis needs to be performed on spikes and bursts that are independent of their neighbors. We used the ISI interval distribution to extract a criterion: events were considered independent if the ISIs followed an exponential distribution [14]. This constrained the ISIs to longer values for bursts (about 300 ms) than for spikes (about 100 ms) and was slightly different for each regime. An ‘event’ was defined as either an isolated single spike or as the first spike in a burst, excluding all following spikes in that burst.

The injected current, membrane potential traces and extracted spikes and bursts can be found here.

### Analysis

#### Cross-correlation

Cross-correlations were calulated over at least three repetitions of 300 s spike trains obtained from five different neurons. A trace at -80 mV was used as the reference spike train. The cross-correlations were not normalized, so the amplitudes in the graphs correspond to the number of coincident spikes.

#### Impedance

The frequency dependent cell impedance Z(f) relates the output membrane potential to the injected input current and can be calculated as the ratio of the absolute values of the Fourier transformed (FFT) output and input signals [15]:

$$Z\left(f\right)=\frac{\left|\text{FFT}(V_{m}\left(t\right))\right|}{\left|\text{FFT}(I_{in}\left(t\right))\right|}$$ (2)

As we were mainly interested in the subthreshold impedance, we removed the depolarizing events from the signal. In a window of 6 ms around each spike and a window of 100-400 ms around each burst the signal was interpolated. Leakage in the fourier transform was prevented by using a Blackman-Harris window.

#### Coherence

Coherence between the input current and the output voltage was calculated using the ‘coherencycpt’ function of the Chronux toolbox (http://chronux.org/, [20]). We used a Jackknife method to approximate the confidence intervals; error bars indicate a p-value of 0.05 (12 tapers with a time-bandwidth product of 10). Coherence was calculated from the first 500 s of the 900 s traces cut into 20 equal parts.

#### Information transfer

The information transfer of a train containing either isolated single spikes, bursts only, events (the combination of the previous two categories or all spikes present, was calculated following [8, 21, 22]: the Fourier transform of the optimal linear filter $\left({\tilde{F}}_{o}\left(f\right)\right)$ was defined as the ratio of the Fourier transform of the cross-correlation between the spike train and the stimulus and the Fourier transform of the autocorrelation of the spike train:

$${\tilde{F}}_{o}\left(f\right)=\frac{\left\langle \tilde{s}\left(f\right)\sum_{j}\;\text{exp}\left(-ift_{j}\right)\right\rangle }{\left\langle |\sum_{j}\;\text{exp}\left(-ift_{j}\right){|}^{2}\right\rangle }$$ (3)

where $\tilde{s}\left(f\right)$ denotes the Fourier transform of the input signal *s*(*t*), and the set {*t*_*j*_} are the spike times. With the help of both causal and acausal parts of this filter the stimulus was reconstructed. The small acausal part was needed because the decision whether a burst or a spike will be fired can sometimes a be made after or during the first spike. To avoid overfitting, the first half of a spike train was used to construct the optimal linear filter, whereas the second half was used for the reconstruction and information estimation. In order to estimate the signal-to-noise ratio $\left(\text{S}\tilde{\text{N}}\text{R}\left(f\right)\right)$ we first estimated the noise level (n(*t*)) by subtracting the reconstructed input (*s*_est_(*t*)) from the injected input (s(*t*)):

$$n\left(t\right)=s\left(t\right)-s_{\text{est}}\left(t\right)$$ (4)

The signal-to noise ration is then defined as

$$\text{S}\tilde{\text{N}}\text{R}\left(f\right)=\frac{\left\langle |\tilde{s}\left(f\right){|}^{2}\right\rangle }{\left\langle |\tilde{n}\left(f\right){|}^{2}\right\rangle }-1$$ (5)

The substraction of −1 comes from the fact that we do not use the estimated signal, but the actual input signal. Finally, the transmitted information rate (*R*(*f*)) as a function of the frequency *f* is given by

$$R\left(f\right)={\text{log}}_{2}\left(1+\text{S}\tilde{\text{N}}\text{R}\left(f\right)\right)$$ (6)

R(f) can be divided by the total number of events to obtain the information rate per event in this frequency band. For smoothening the power spectrum densities we used the Welch’s averaged modified periodogram method that is standard in MATLAB.

#### ETA and ETC

Event-Triggered Covariance analysis (ETC) was used to determine what features the TCR cell extracts from the input [23, 25, 26]. The input current *s*(*t*) is sampled (2 kHz) in a time-window from 180 ms before to 50 ms after the independent event. This set of sampled input currents ${\vec{s}}_{n}$ around all event-times forms the event-triggered ensemble and the mean of this ensemble ${\langle {\vec{s}}_{n}\rangle }_{n}$ is the event-triggered average (ETA). The second moment of this ensemble is the covariance. The elements of the covariance matrix are defined as the covariances between different time-points *τ*_*i*_ around the stimulus [24, 25, 27], so of different elements of the vector $\vec{s}$:

$$C_{ij}={\left\langle \left(s_{n}\left(\tau_{i}\right)-{\left\langle s_{k}\left(\tau_{i}\right)\right\rangle }_{k}\right)\left(s_{n}\left(\tau_{j}\right)-{\left\langle s_{k}\left(\tau_{j}\right)\right\rangle }_{k}\right)\right\rangle }_{n},$$ (7)

where 〈*s*_*k*_(*τ*_*i*_)〉_*k*_ is the mean over all event-times *t*_*k*_ of a single time-point *τ*_*i*_ in the window around the event. Assuming that the covariance of stimuli that trigger an event is different from the covariance of the total input current (the prior, constructed as the same number of sampled time-windows, but seeded at random points), we can look at the directions in the multidimensional space in which the covariance changes (the dimension of the space is the amount of sample-points in the time-window around the event). This provides information about features in the input signal that triggered events. The covariance difference matrix was constructed by subtracting the prior covariance matrix from the event-triggered covariance (ETC) matrix:

$${\hat{C}}_{ij}=C_{ij}-C_{ij}^{\text{prior}}$$ (8)

The eigenvectors ${\vec{v}}_{i}$ of $\hat{C}$ belonging to the largest or smallest eigenvalues span the subspace with the largest difference between the ETC and the prior covariance. Therefore, these eigenvectors or filters (or any linear combination of them) demonstrate to which features in the input signal the neuron is sensitive. The relative contribution of a filter to event generation, can be calculated from a non-linear threshold function or decision function for that filter, $P(event|{\vec{v}}_{i})$ using Bayes law as:

$$P\left(event|{\vec{v}}_{i}\right)=\frac{P\left({\vec{v}}_{i}|event\right)P\left(event\right)}{P({\vec{v}}_{i})}$$ (9)

in which $P({\vec{v}}_{i}|event)$ is the event-triggered ensemble projected onto filter ${\vec{v}}_{i}$, *P*(*event*) is the mean firing rate over the entire period and $P({\vec{v}}_{i})$ is the prior distribution projected onto filter ${\vec{v}}_{i}$ [24, 27]. $P({\vec{v}}_{i}|event)$ and $P({\vec{v}}_{i})$ can be sampled to estimate $P(event|{\vec{v}}_{i})$. Note that for both the ETA and the ETC these projections are onto normalized (L2 norm) filters. The decision function can be generalized to multiple dimensions in order to construct a linear-nonlinear model with multiple filters:

$$P\left(event|{\vec{v}}_{i},\ldots ,{\vec{v}}_{j}\right)=\frac{P\left({\vec{v}}_{i},\ldots ,{\vec{v}}_{j}|event\right)P\left(event\right)}{P({\vec{v}}_{i},\ldots ,{\vec{v}}_{j})}$$ (10)

### Model

#### Model equations

This study uses the well-validated three-compartment model of Destexhe et al. [12] that covers the basic properties of the TCR cell, such as a shift from a bursting to a spiking regime and earlier spiking with depolarization. It is a reduction to three compartments (soma, proximal dendrite and distal dendrite) of a multi-compartment model of a rat dissociated thalamocortical relay cell. The soma contains sodium current, potassium current, leak current and T-type calcium current, whereas the dendrites only contain leak current and T-type calcium current. All compartments contain calcium accumulation as a result of calcium currents and a simple exponential decay (*τ* = 5 ms) that restores the *Ca*^2+^ concentration back to a uniform resting level of 200 nM (the extracellular *Ca*^2+^ concentration is kept at 2 mM). Three currents were added: an h-current *I*_*h*_ (${\bar{g}}_{h}=130$ *μS*/cm^2^) with a kinetic model described in [32, 33], a high-threshold calcium current *I*_*L*_ (${\bar{p}}_{L}=0.00005$ *cm*/*s*) and a *Ca*^2+^ activated *K*^+^ current *I*_*KC*_ (${\bar{g}}_{KC}=1000$ *μS*/cm^2^). The last two written by Arthur Houweling after the book *Electrophysiology of the Neuron* [31]. All can be found on the ModelDB (accession numbers 279, 3343 and 3808, https://senselab.med.yale.edu/ModelDB). The model was implemented in Neuron [29] with an integration time step of 0.1 ms.

The high-conductance state was implemented following [35] (accession number 8115): a membrane current resulting from independently fluctuating passive inhibitory and excitatory conductances was implemented in the soma.

$$\begin{array}{c} \begin{array}{cc} I_{\text{fluct}} & =g_{e}\left(V_{m}-E_{e}\right)+g_{i}\left(V_{m}-E_{i}\right)\\ \frac{dg_{e}}{dt} & =\frac{-(g_{e}-g_{e0})}{\tau_{e}}+\chi_{1}\left(t\right)\sqrt{D_{e}}\\ \frac{dg_{i}}{dt} & =\frac{-(g_{i}-g_{i0})}{\tau_{i}}+\chi_{2}\left(t\right)\sqrt{D_{i}}, \end{array} \end{array}$$ (11)

where *E*_*e*_ = 0 mV and *E*_*i*_ = −80 mV are the reversal potentials of the excitatory and inhibitory currents and *χ*(*t*) is Gaussian white noise with parameters *D* = 2*σ*^2^/*τ*, *σ*_*e*_ = 3.0 nS, *σ*_*i*_ = 6.6 nS, *τ*_*e*_ = 2.7 ms and *τ*_*i*_ = 10.5 ms. In the ‘standard’ high-conductance state, *g*_*e*0_ = 12.1 nS and *g*_*i*0_ = 57.3 nS. In the ‘inhibitory’ high conductance state, the total conductance was left the same, but now all conductances were inhibitory: *g*_*i*0_ = 70 nS. In order to keep the width of the membrane potential distribution comparable between the standard and the inhibitory high-conductance state, in the inhibitory case the standard deviation of the fluctuation was increased to *σ*_*i*_ = 10 nS. The fluctuating background conductance was not strong enough to make the model neuron spike. Therefore, an exponential synapse (conductance: 100 nS, reversal potential 0 mV, *τ* = 3 ms) was added to the proximal dendrite, activated by a Poisson spike train with a mean ISI of 100 ms (‘Randomstream’ template, [36, 37], modeldB entry 83319). In the case with synchronized inhibitory input, an inhibitory synapse (conductance: 300 nS, reversal potential -80 mV, *τ* = 10 ms) was added to the proximal dendrite. This inhibitory synapse was activated by the same Poisson spike train as the excitatory synapse, but shifted 35 ms earlier in time.

The model code can be found in the ModelDB, accession number 232876.

#### Model input

The model was activated by somatic current injection that was identical to the one used in the experiments, be it scaled to an amplitude that induced the same voltage fluctuations as observed in the experiments. The DC-component was adjusted so that the average subthreshold membrane potential was comparable to those in the experiments. This resulted in an input current with a standard deviation of *σ* = 100 pA for case 1, and a mean of −700 pA, −475 pA, −275 pA and 0 pA for membrane states of respectively −80 mV, −70 mV, −60 mV and −50 mV. Since the model is deterministic, an additional noise source is needed to assess the sensitivity of the neuron to small changes in the input. To allow a comparison between the model and the experiments we added trial-to-trial variability for the short 300 s traces: to each trial a DC component was added that was drawn from a Gaussian distribution with a mean of *μ* = 0 pA and a standard deviation of *σ* = 50 pA and white noise with a mean of *μ* = 0 pA and a standard deviation of *σ* = 2 pA.

#### Reliability

The validity of the model was assessed by comparing its spike times with those of the neuron when exposed to the same input current. In the ideal situation the reliability between a model trace and an experimental trace should be comparable with those between experimental traces from different cells. To this end, a spike train is reduced to a series of delta pulses:

$$s_{i}\left(t\right)=t_{i1},t_{i2},\ldots ,t_{iN_{i}}=\sum_{m=1}^{N_{i}}\delta \left(t-t_{im}\right)$$ (12)

Hunter and Milton [68] define reliability between two spike trains *s*_*i*_(*t*) and *s*_*j*_(*t*) as

$$R_{HM}=\frac{1}{2}\left(\left\langle r_{ij}\right\rangle +\left\langle r_{ji}\right\rangle \right),$$ (13)

where

$$\left\langle r_{ij}\right\rangle =\frac{1}{N_{i}}\sum_{k=1}^{N_{i}}\text{exp}\left(-\frac{\Delta t_{k}}{\tau_{HM}}\right)$$ (14)

and Δ*t*_*k*_ is the absolute value of the difference between spike time *t*_*k*_ in spike train *s*_*i*_ and the nearest neighbour spike time in spike train *s*_*j*_. We also compared spike trains using the coincidence factor [69, 70]. This measure is based on the binning of the spike train in $K=\frac{T}{p}$ bins of precision *p*. It is defined as

$$\Gamma =\frac{N_{\text{coinc}}-\left\langle N_{\text{coinc}}\right\rangle }{\frac{1}{2}\left(N_{i}+N_{j}\right)}\frac{1}{N},$$ (15)

in which

$$\left\langle N_{\text{coinc}}\right\rangle =2f_{j}pN_{i}=\frac{2N_{i}N_{j}}{K}=2f_{i}f_{j}pT$$ (16)

So it is corrected for the expected number of coincidences 〈*N*_coinc_〉 of spike train *s*_*i*_ with a Poissonian spike train with the same rate *ν*_*j*_ as spike train *s*_*j*_. Γ is normalized by N

$$N=1-2\frac{\text{max}(N_{i},N_{j})p}{T}$$ (17)

The normalization is in the original formulation not done by the maximum of *N*_*i*_ and *N*_*j*_, but by one of them, we used the maximum. It is 1 for identical spike trains, 0 when all coincidences are accidental and negative for anti-correlated spike trains.

The difference between the model and the experimental spike trains relative to the variability of experimental spike trains was quantified as follows: the reliability (Γ or *R*_*HM*_) between experimental traces *R*_*e*−*e*_ was calculated at a precision of 10 ms to create a null-distribution *P*_0_(*R*_*e*−*e*_). Next, the surface of the null-distribution for every reliability value between experimental and model traces *R*_*m*−*e*_ was calculated at a precision of 10 ms:

$$S_{m-e,0}=\int_{-\infty }^{R_{m-e}}P_{0}\left(x\right)dx$$ (18)

For values of *R*_*m*−*e*_ drawn from the null-distribution *P*_0_(*R*_*e*−*e*_), the expectation value of *S*_*m*−*e*,0_ is 0.5: i.e. the model spike trains were as (dis)similar to the experimental spike trains as the experimental spike trains are amongst each other. If the mean of the distribution of *S*_*m*−*e*,0_-values is smaller than 0.5, the model spike trains are less similar to the experimental spike trains than the experimental spike trains are to each other. Fig 10 provides the mean and standard deviation (error bars) of *S*_*m*−*e*,0_. We compared the (null-)distributions of the reliability values of spike trains from the same cell only with those of spike trains from different cells only. The reliability values of spike trains from the same cell were clearly higher, but the (null-)distribution of the reliability values of spike trains from different cells only, was hardly different from the distribution of the reliability values of all spike trains. There fore all spike trains, whether they were from the same cell or from different cells were included. At each holding potentials of −80,−70 and −60 mV we had three traces of 300 s from five cells, so 15 traces and 90 comparisons in total. For a holding potential of −50 mV we had only two cells and nine comparisons. For comparing model traces with experimental traces, we had 15 simulated traces for each holding potential, resulting in 90 comparisons at −50 mV and 225 comparisons at the other states.

We are aware of several problems with this kind of comparison. Firstly, it depends on the overall frequency [34]. Therefore, we will compare each regime separately. Secondly, a trial-by-trial comparison between model and experimental spike trains might result in a deterministic bias [71]. Because we are not interested here in comparing deterministic with probabilistic models, we did not include a correction.

## Supporting information

S1 Fig Reliability of event times between cells. Since all analyses in this paper critically depend on spike timing, the differences in spike timing show directly the consistency of our results on a population level. In supplementary figure 1 we compare the reliability of spike timing between recordings within the same cell (blue traces), between recordings of different cells (green traces) and between recordings and simulations (red traces), at a precision of 10 ms. We used the 300 s. recordings (repeated 3 times, see Materials and methods and our paper [34]).
(TIF)

S2 Fig ETAs of two different cells. ETAs for two cells (red and black) from S1 Fig, and for a Poisson event-train with the same number of events as the red trace. Even though the reliability between the cells of which we show the ETAs is quite low, the ETAs are still very comparable.
(TIF)

S3 Fig Eigenvalues of the covariance analysis (Fig 8). (TIF)

S4 Fig Bias of information calculation. The same analysis as in Fig 5 repeated 50 times, but using Poisson event-trains with the same number of events as in Fig 5. Error-bars denote standard deviations.
(TIF)
