# Supplementary figures and images for: Spike and burst coding in thalamocortical relay cells
Source: PLoS Comput Biol. 2018 Feb 12;14(2):e1005960. doi: 10.1371/journal.pcbi.1005960 (PMC5834212; doi:10.1371/journal.pcbi.1005960)

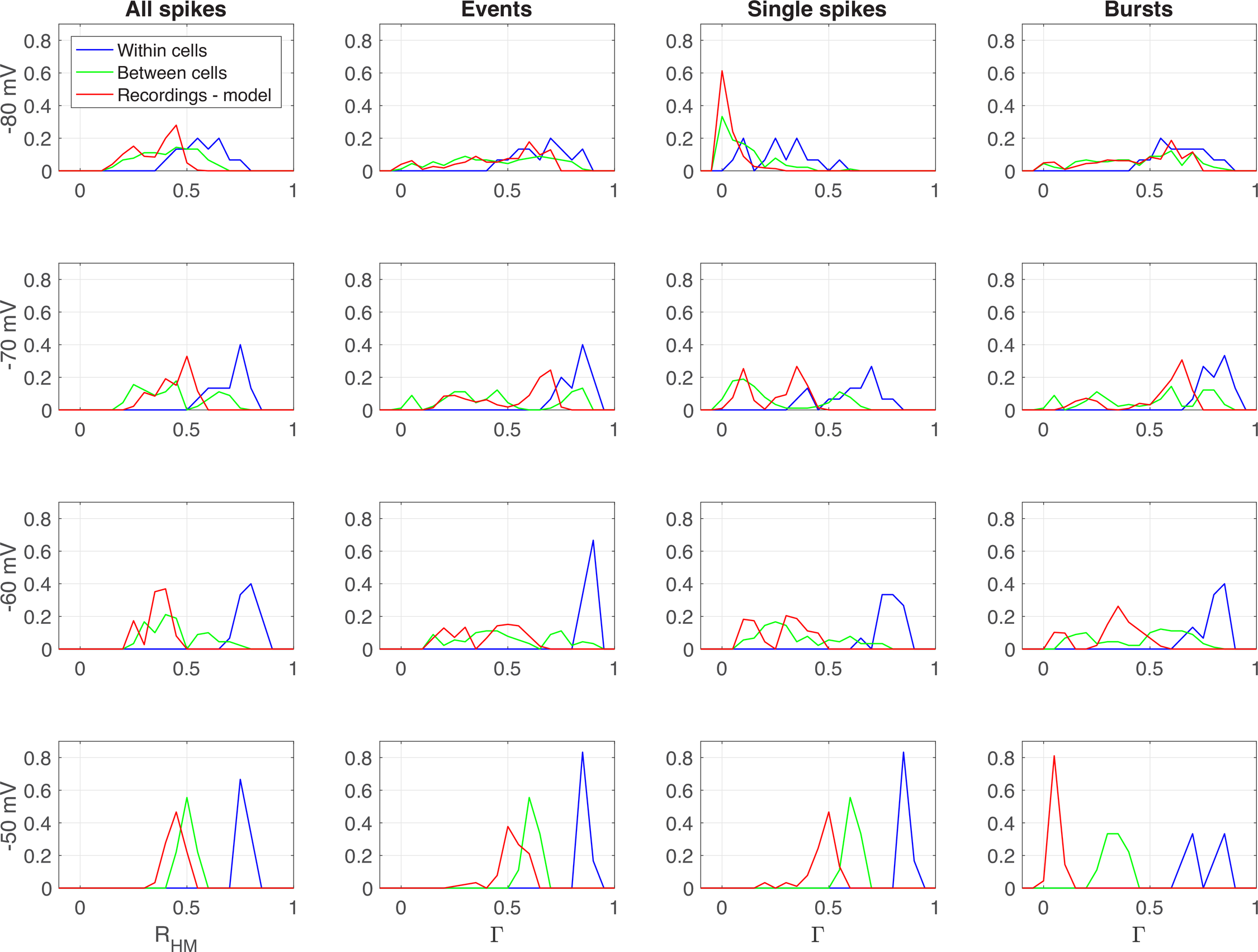

Supplement: S1 Fig — Since all analyses in this paper critically depend on spike timing, the differences in spike timing show directly the consistency of our results on a population level. In supplementary figure 1 we compare the reliability of spike timing between recordings within the same cell (blue traces), between recordings of different cells (green traces) and between recordings and simulations (red traces), at a precision of 10 ms. We used the 300 s. recordings (repeated 3 times, see Materials and methods and our paper [34]). (TIF) [file pcbi.1005960.s001.tif]

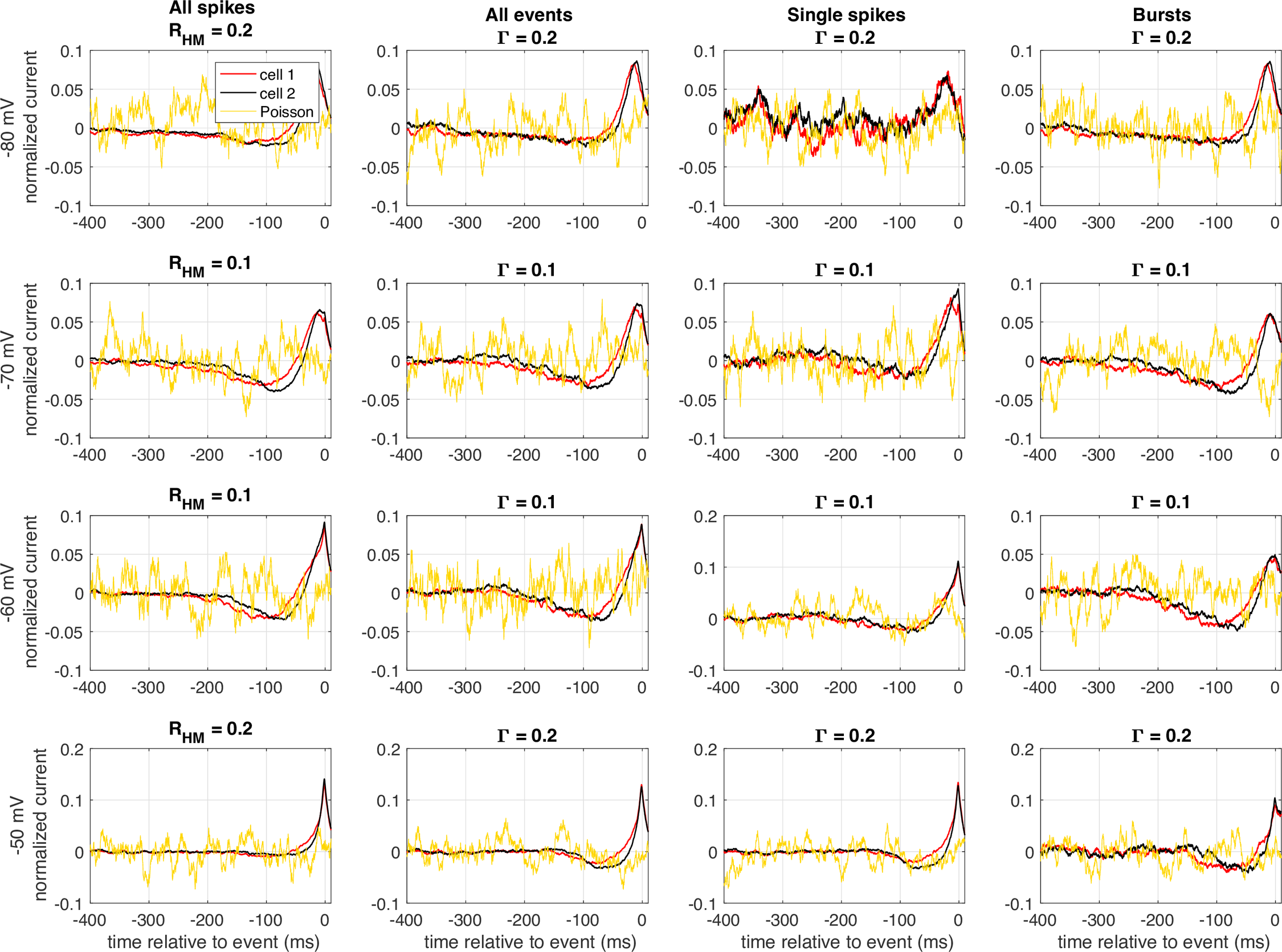

Supplement: S2 Fig — ETAs for two cells (red and black) from S1 Fig, and for a Poisson event-train with the same number of events as the red trace. Even though the reliability between the cells of which we show the ETAs is quite low, the ETAs are still very comparable. (TIF) [file pcbi.1005960.s002.tif]

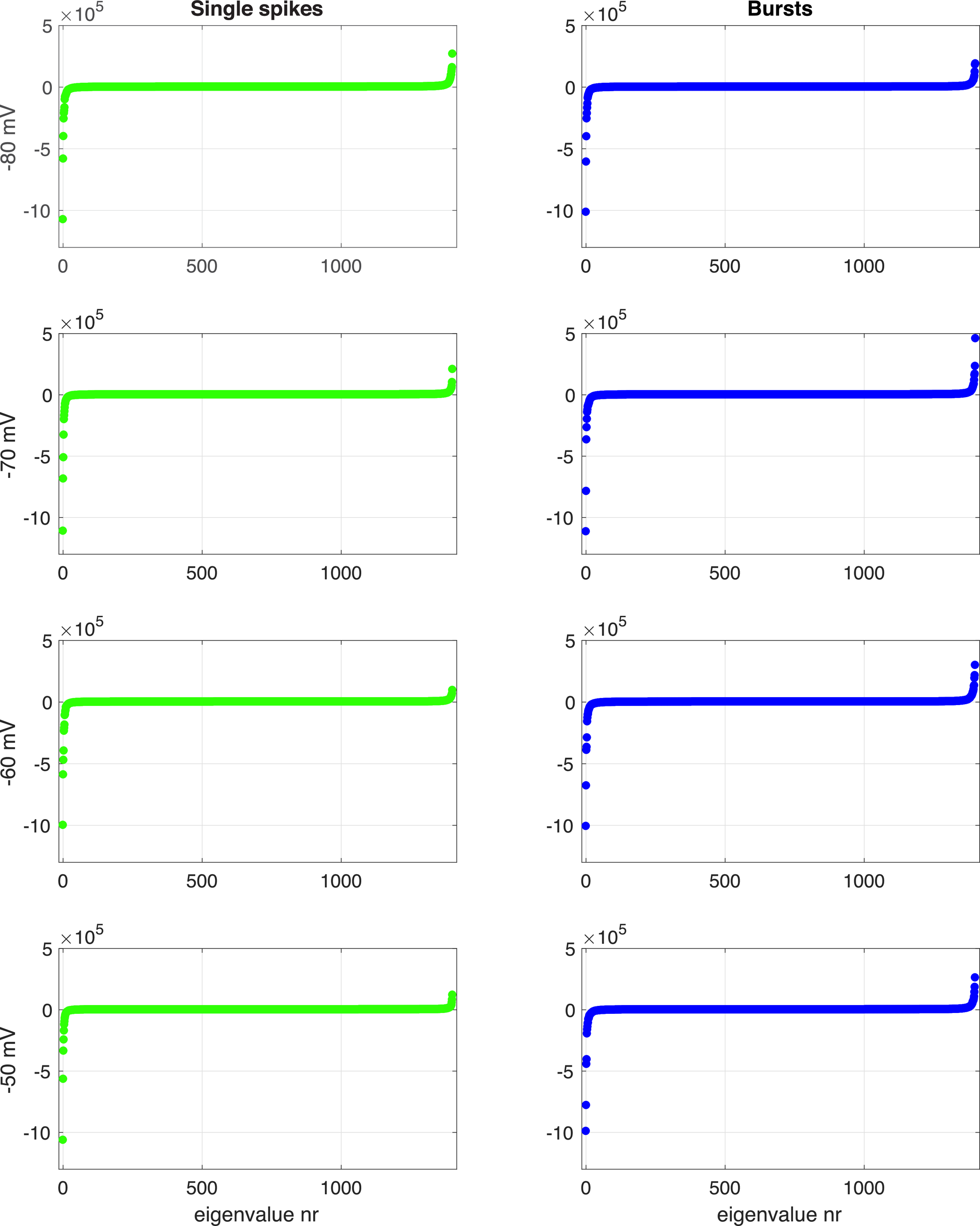

Supplement: S3 Fig — (TIF) [file pcbi.1005960.s003.tif]

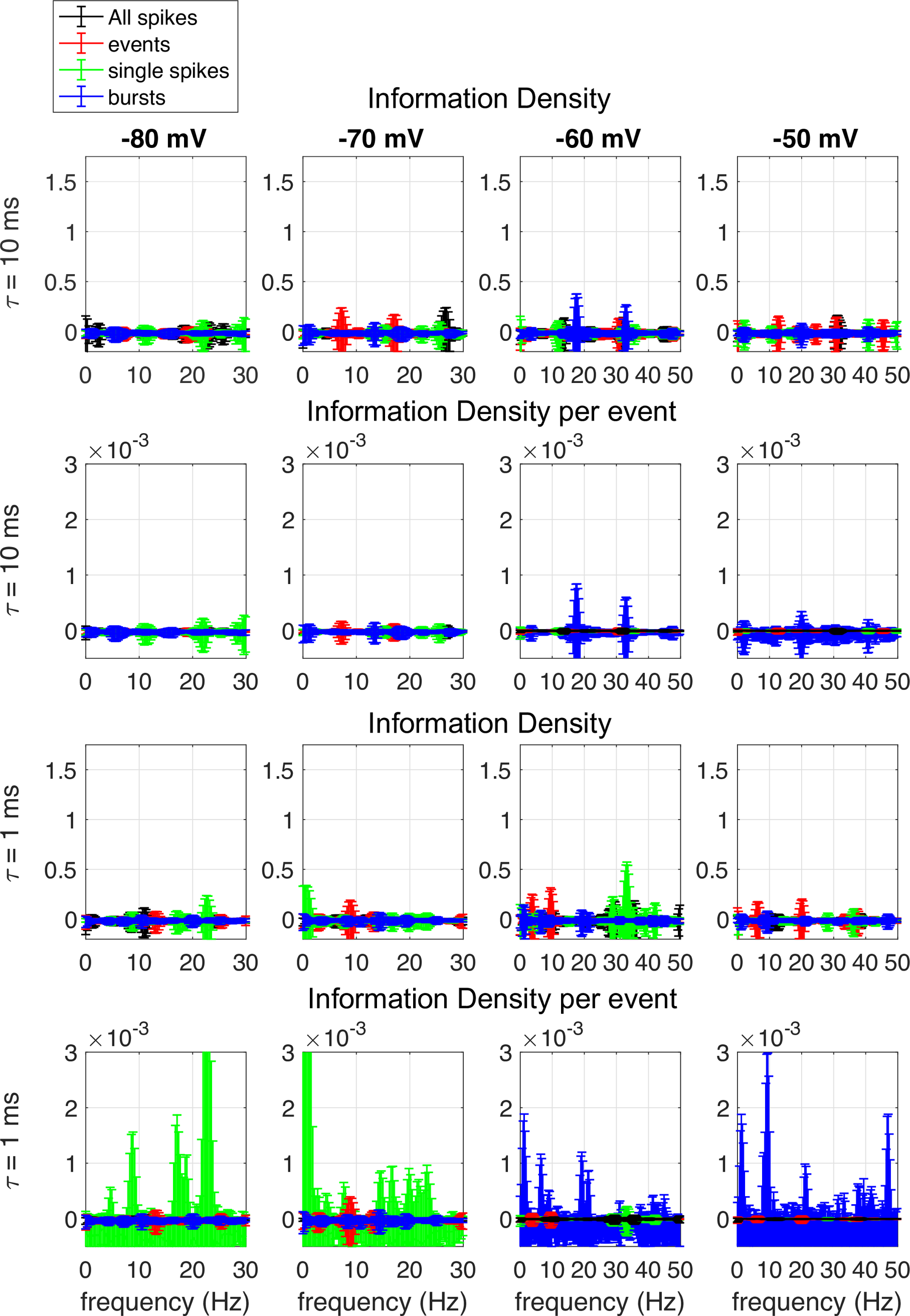

Supplement: S4 Fig — The same analysis as in Fig 5 repeated 50 times, but using Poisson event-trains with the same number of events as in Fig 5. Error-bars denote standard deviations. (TIF) [file pcbi.1005960.s004.tif]
